# Lifestyle Medicine as Co-Therapy During Incretin-Based Anti-Obesity Pharmacotherapy: Integrating Physical Activity, Nutrition, and Behavioral Strategies for Long-Term Success

**DOI:** 10.3390/nu18172748

**Published:** 2026-08-22

**Authors:** Marta Mallardo, Antonietta Messina, Vincenzo Monda, Marco La Marra, Antonietta Monda, Salvatore Allocca, Maria Casillo, Girolamo Di Maio, Pasquale Perrone, Aurora Daniele, Marcellino Monda, Giovanni Messina, Fiorenzo Moscatelli, Rita Polito

**Affiliations:** 1 Department of Education and Sport Sciences, Pegaso Telematic University, 80143 Naples, Italy; marta.mallardo@unipegaso.it (M.M.); fiorenzo.moscatelli@unipegaso.it (F.M.); 2 Department of Precision Medicine, University of Campania “Luigi Vanvitelli”, 80138 Naples, Italy; antonietta.messina@unicampania.it; 3 Department of Economics, Law, Cybersecurity, and Sports Sciences, University of Naples “Parthenope”, 80131 Naples, Italy; vincenzo.monda@uniparthenope.it; 4 Section of Human Physiology, Unit of Dietetics and Sports Medicine, Department of Experimental Medicine, University of Campania “Luigi Vanvitelli”, 80138 Naples, Italy; marco.lamarra@unicampania.it (M.L.M.); salvatore.allocca@unicampania.it (S.A.); maria.casillo@unicampania.it (M.C.); marcellino.monda@unicampania.it (M.M.); 5 Department of Human Science and Quality of Life Promotion, San Raffaele Telematic University, 00166 Rome, Italy; antonietta.monda@uniroma5.it; 6 Department of Psychology and Health Sciences, Pegaso Telematic University, 80143 Naples, Italypasquale.perrone@unipegaso.it (P.P.); rita.polito@unipegaso.it (R.P.); 7 Department of Molecular Medicine and Medical Biotechnology, University of Naples Federico II, Via Sergio Pansini 5, 80131 Naples, Italy; aurora.daniele@unina.it

**Keywords:** obesity pharmacotherapy, incretin-based therapies, GLP-1 receptor agonists, tirzepatide, physical activity, nutritional strategies, lean mass preservation, weight-loss maintenance

## Abstract

Background/Objectives: Obesity is a chronic, progressive, and relapsing disease that requires long-term, multidisciplinary management rather than episodic weight-loss treatment. Although novel incretin-based anti-obesity pharmacotherapies, including GLP-1 receptor agonists and dual GIP/GLP-1 receptor agonists, have markedly improved the clinical management of obesity, weight reduction alone does not fully capture treatment success. Body composition, lean mass preservation, physical function, nutritional adequacy, psychological well-being, adherence, and long-term weight-loss maintenance are increasingly recognized as essential therapeutic outcomes. This narrative review critically examines the role of lifestyle medicine as a co-therapeutic strategy during modern anti-obesity pharmacotherapy, with particular attention to physical activity, nutrition, behavioral support, and individualized monitoring. Methods: A narrative literature search was conducted in PubMed up to June 2026. The review included studies addressing adults with overweight or obesity and evidence related to anti-obesity pharmacotherapy, physical activity, nutrition, body composition, functional outcomes, eating behavior, quality of life, treatment tolerability, adherence, weight regain, and long-term maintenance. Results: Current evidence indicates that incretin-based therapies produce substantial and clinically meaningful weight loss, but pharmacological efficacy may be limited by reductions in lean mass, gastrointestinal adverse events, inadequate nutritional intake, treatment discontinuation, and weight regain after drug withdrawal. Physical activity should be considered a therapeutic component rather than only a tool for increasing energy expenditure, as aerobic exercise supports cardiometabolic health and cardiorespiratory fitness, while resistance training helps preserve muscle strength, bone health, and functional capacity. Nutritional strategies are equally important, particularly during appetite suppression, to maintain adequate protein, fiber, fluids, micronutrients, and diet quality. Behavioral factors, including sleep, stress, mood, stigma, self-regulation, and the food environment, may influence adherence and long-term outcomes. Conclusions: Novel anti-obesity drugs should not be viewed as replacements for lifestyle medicine but as powerful tools within an integrated chronic-care model. The goal of treatment should move beyond maximal body-weight reduction to durable improvements in body composition, metabolic health, physical function, nutritional status, quality of life, and weight-loss maintenance.

## 1. Introduction

Obesity is recognized as a chronic, progressive, and relapsing disease that requires long-term management rather than episodic weight-loss treatment [1]. Its worldwide prevalence has more than doubled since 1990, with approximately 890 million adults living with obesity in 2022; this trend contributes substantially to the global clinical and economic burden of non-communicable diseases [2,3]. Beyond excess adiposity, obesity is associated with multiple complications, including cardiovascular disease, type 2 diabetes, gastrointestinal and respiratory disorders, musculoskeletal impairment, psychological distress, reduced quality of life, and increased mortality risk [4,5,6,7]. Thus, modern obesity treatment demands a multidisciplinary, individualized, and long-term approach to the treatment of obesity that focuses on both biological and behavioral factors [6]. Lifestyle changes remain the cornerstone of obesity treatment. However, traditional methods that are based mainly on caloric restriction (CR) and general lifestyle advice are not likely to produce lasting effects. Factors such as low adherence rates, lack of access to specialist professionals, and lack of support can reduce the effectiveness of these conventional methods [8,9]. These limitations do not reduce the importance of lifestyle interventions; rather, they highlight the need to move from isolated lifestyle advice toward structured lifestyle medicine, in which nutritional strategies, physical activity (PA), behavioral support, and long-term monitoring are integrated into chronic obesity care.

In parallel, the development of new and promising anti-obesity pharmacotherapies, particularly incretin-based agents, has expanded treatment options and improved metabolic outcomes [10,11]. Glucagon-like peptide-1 (GLP-1) receptor agonists and dual glucose-dependent insulinotropic polypeptide/glucagon-like peptide-1 (GIP/GLP-1) receptor agonists act on central and peripheral neuroendocrine pathways involved in appetite regulation, satiety, glucose homeostasis, and energy balance [10]. These mechanisms have enabled clinically meaningful weight reduction and improvements in obesity-related metabolic risk factors, reshaping the pharmacological management of obesity.

However, the increasing efficacy of anti-obesity pharmacotherapy has also raised new clinical questions. The benefits of weight loss on body composition, skeletal muscle function, cardiorespiratory fitness, nutritional intake, psychological health, and weight-loss maintenance are not necessarily optimal. Pharmacologically induced weight loss can result in a reduction in fat-free mass, which can include lean tissue components, and may be more clinically significant in older people, in those with sarcopenic obesity, and in patients with low baseline physical function [11,12]. Moreover, gastrointestinal adverse events, reduced food intake, treatment discontinuation, weight regain after withdrawal, and inter-individual variability in behavioral responses may limit the durability and real-world effectiveness.

Within this context, PA and nutritional strategies should be considered essential complementary strategies rather than alternatives to pharmacotherapy [13]. Accordingly, aerobic exercise may improve cardiorespiratory fitness, insulin sensitivity, vascular function, mitochondrial metabolism, and cardiometabolic health, whereas resistance training is particularly relevant for preserving skeletal muscle mass, strength, bone health, and functional capacity during weight reduction [12,14]. In addition, non-exercise activity thermogenesis (NEAT) and reductions in sedentary behavior may contribute to daily energy expenditure and support long-term weight-loss maintenance [15]. Similarly, nutritional strategies are equally important during pharmacological weight loss, especially when appetite suppression may reduce total food intake and increase the risk of inadequate nutrient consumption [16,17].

Another relevant dimension regards the interaction between pharmacological treatment, eating behavior, and lifestyle-related neurobehavioral mechanisms. Novel anti-obesity drugs act partly through central pathways involved in appetite, satiety, food reward, and eating control [17]. Sleep, stress, mood, PA, dietary patterns, food environment, stigma, and mental health are also factors that affect eating behavior. Therefore, long-term treatment success likely requires more than drug-induced appetite suppression and should include behavioral adaptation, adherence support, and sustainable lifestyle change.

Although both novel anti-obesity pharmacotherapies and lifestyle interventions have been extensively investigated, evidence regarding how structured lifestyle strategies can be optimally integrated with these medications, and their additional effects during pharmacological treatment, is still changing. Recent reviews of pivotal anti-obesity pharmacotherapy trials indicate that efficacy assessments have focused primarily on body-weight reduction and cardiometabolic outcomes [8,9], whereas the integration of structured lifestyle interventions and the evaluation of body composition, physical function, exercise capacity, nutritional adequacy, treatment adherence, weight-loss maintenance, and patient-centered outcomes have been less consistent [13,16]. This gap is clinically relevant because obesity is shaped by biological, psychological, social, and behavioral determinants, and individuals living with obesity often experience stigma, weight bias, impaired quality of life, and treatment-related burden [18,19].

**Figure 1:**
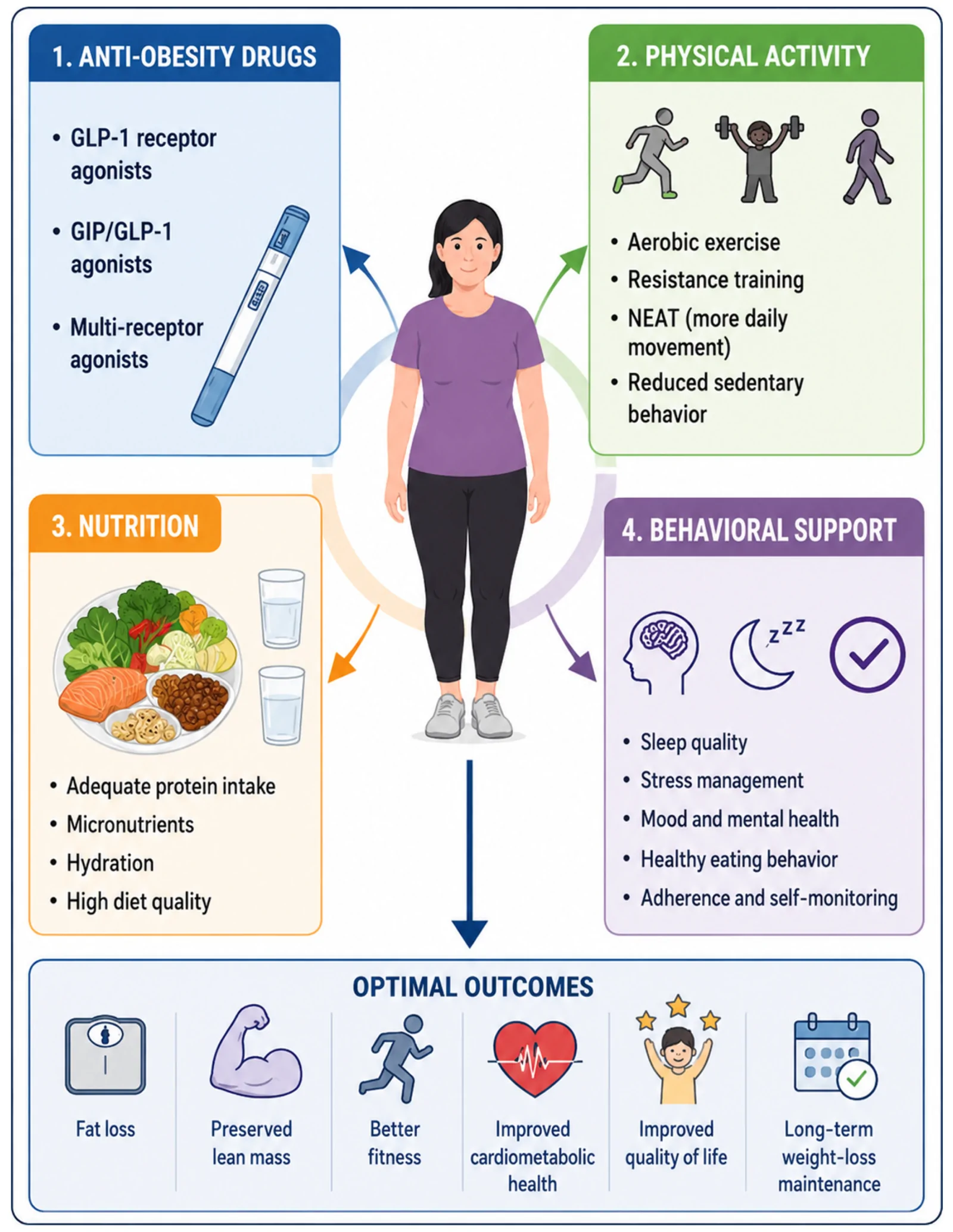
Integrated lifestyle co-therapy during novel anti-obesity pharmacotherapy. Incretin-based and emerging anti-obesity pharmacotherapies induce clinically meaningful weight loss whilst structured lifestyle medicine may help optimize body composition, physical function, cardiometabolic health, quality of life, and long-term weight-loss maintenance.

In this scenario, this review aims to critically examine how structured lifestyle interventions, including PA, nutritional strategies, behavioral support, and individualized monitoring, may complement novel anti-obesity pharmacotherapies. Specifically, it discusses how lifestyle-based co-therapy may optimize body composition, cardiometabolic health, physical function, health-related quality of life, and long-term weight-loss maintenance, supporting a more integrated and patient-centered model of obesity care. The conceptual framework for this integrated model, highlighting the synergy between pharmacotherapy and lifestyle factors, is summarized in Figure 1.

## 2. Methods

This article was designed as a narrative review aimed at critically discussing the role of lifestyle medicine as a co-therapeutic strategy during incretin-based and emerging anti-obesity pharmacotherapy. The review focuses on physical activity, nutritional strategies, behavioral factors, body composition, functional outcomes, quality of life, and long-term weight-loss maintenance.

Relevant literature was identified through PubMed searches up to June 2026.

The principal search terms included “obesity”, “overweight”, “anti-obesity medication”, “pharmacological weight loss”, “GLP-1 receptor agonist”, “liraglutide”, “semaglutide”, “tirzepatide”, “retatrutide”, “dual agonist”, and “multi-receptor agonist”, combined using the Boolean operator AND with terms including “physical activity”, “exercise”, “aerobic exercise”, “resistance training”, “sedentary behavior”, “non-exercise activity thermogenesis”, “nutrition”, “diet”, “protein intake”, “body composition”, “lean mass”, “muscle mass”, “eating behavior”, “mental health”, “quality of life”, “weight regain”, and “weight-loss maintenance”. Eligible publications were peer-reviewed articles written in English that: (i) included adults with overweight or obesity receiving incretin-based or emerging anti-obesity pharmacotherapy; (ii) examined lifestyle factors directly relevant to pharmacological weight management, including physical activity, exercise, nutrition, eating behavior, or psychological and behavioral support; or (iii) reported outcomes concerning body composition, cardiometabolic health, physical function, quality of life, treatment discontinuation, or long-term weight maintenance. Because direct evidence combining modern anti-obesity medications with structured lifestyle interventions remains limited, studies evaluating lifestyle strategies without concomitant pharmacotherapy were also considered when they provided clinically relevant evidence regarding muscle preservation, functional capacity, dietary adequacy, behavioral adaptation, or weight-loss maintenance.

Studies were excluded if they (i) were conducted exclusively in animals or in vitro; (ii) focused on pediatric populations; (iii) evaluated pharmacological treatments unrelated to obesity management; (iv) did not address any of the lifestyle, clinical, functional, or behavioral outcomes relevant to the scope of the review; or (v) were conference abstracts, study protocols, editorials, commentaries, or publications for which the full text was unavailable. The evidence synthesis prioritized original studies, particularly randomized controlled trials, extension and withdrawal studies, and body-composition studies, together with systematic reviews and meta-analyses.

Evidence was narratively synthesized according to the main domains of the review: pharmacological efficacy and limitations, physical activity as co-therapy, nutritional strategies during appetite suppression, neurobehavioral interactions, clinical outcomes beyond body weight, and long-term maintenance.

## 3. Novel Anti-Obesity Pharmacotherapies: Clinical Context and Implications for Lifestyle Co-Therapy

Pharmacologic therapy of obesity has undergone significant changes since the introduction of incretin-based drugs. Until recently, the drugs that have been available have resulted in modest weight loss and long-term poor adherence and safety or tolerability issues. Glucagon-like peptide-1 receptor agonists and dual incretins, on the other hand, have been able to achieve weight loss rates comparable to those previously seen primarily following bariatric surgery, and have shown improvements in a number of obesity-related metabolic parameters. Currently, semaglutide and tirzepatide are two of the most successful medications for chronic weight management, and obesity is a chronic condition that needs chronic and comprehensive care [20]. Their clinical efficacy has further reinforced the concept of obesity as a chronic disease requiring integrated management.

### 3.1. Single GLP-1 Receptor Agonists: Mechanisms and Clinical Context

Glucagon-like peptide-1 receptor agonists represent a major advance in obesity pharmacotherapy. When combined with lifestyle intervention, these agents produce greater weight loss than placebo plus lifestyle intervention in randomized clinical trials [21]. These agents, such as liraglutide and semaglutide, mimic the activity of endogenous GLP-1, an incretin hormone involved in glucose homeostasis, appetite regulation, and energy intake [22]. Their effects are mediated through central and peripheral pathways: centrally, they modulate appetite-related circuits in the brainstem, hypothalamus, and reward systems, thereby reducing hunger and food intake; peripherally, they slow gastric emptying, promote earlier satiation, and improve glycemic regulation [23].

From a clinical point of view, liraglutide is administered once daily, whereas semaglutide is administered once weekly, a difference that may influence adherence, treatment acceptability, and long-term use [24].

The SCALE and STEP clinical trial programs established the efficacy of liraglutide and semaglutide for chronic weight management [25,26,27,28,29]. In addition to body-weight reduction, semaglutide has been associated with improvements in waist circumference, glycemic control, blood pressure, lipid profile, and patient-reported physical functioning [26,30,31]. However, gastrointestinal adverse events, treatment cost, inter-individual variability, and the need for continued treatment may affect long-term persistence [32]. Weight regain after discontinuation further supports the integration of pharmacotherapy with nutritional care, physical activity, and behavioral strategies aimed at long-term maintenance [33].

### 3.2. Dual and Multi-Receptor Agonists: Clinical Context and Lifestyle-Relevant Implications

Dual incretin agonism has extended obesity pharmacotherapy beyond selective GLP-1 receptor targeting. Tirzepatide, a first-in-class dual glucose-dependent insulinotropic polypeptide (GIP) and GLP-1 receptor agonist, has shown greater reductions in body weight and glycemic indices than earlier GLP-1 receptor agonist-based approaches in several clinical settings [34]. The rationale for dual agonism is based on the complementary metabolic actions of GLP-1 and GIP signaling. As discussed above, GLP-1 receptor activation is mainly known to decrease appetite, delay stomach emptying, and enhance glucose-dependent insulin secretion, whereas GIP receptor activation might have additional effects on insulin secretion, adipose tissue metabolism, central appetite control, and energy balance [35,36]. Thus, co-targeting GLP-1 and GIP receptors could potentially have effects that are synergistic for weight loss and glycemic control, but the specific role of each pathway over the long-term in body composition and functional outcomes is not fully characterized.

The SURMOUNT clinical trial program established the efficacy of tirzepatide across different populations and treatment settings. SURMOUNT-1 and SURMOUNT-2 demonstrated substantial weight reduction in adults with obesity or overweight, with and without type 2 diabetes, while SURMOUNT-3 showed additional weight loss following an intensive lifestyle intervention [37,38,39]. In SURMOUNT-5, tirzepatide produced greater reductions in body weight and waist circumference than semaglutide [40]. These findings provide relevant clinical context because the magnitude of weight loss may influence nutritional requirements, body-composition changes, physical function, and the intensity of lifestyle support required during treatment.

In addition to tirzepatide, next-generation multi-receptor agonists are rapidly moving forward, such as triple receptor agonists like retatrutide, which have shown up to 24.2% weight loss at 48 weeks in phase 2 trials, and other dual- and triple-agonist programs in clinical development [41]. Another strategy combines GLP-1 receptor agonism with amylin signaling. Cagrilintide–semaglutide achieved an estimated mean body weight reduction of 20.4% at 68 weeks in the phase 3 REDEFINE 1 trial, compared with 3.0% with placebo [42].

Another emerging strategy is dual GLP-1/glucagon receptor agonism. Survodutide has shown dose-dependent weight reduction in phase 2 obesity trials and has also demonstrated histological benefits in metabolic dysfunction-associated steatohepatitis, suggesting potential relevance for obesity-related hepatic and cardiometabolic complications [43,44]. Similar agents, including mazdutide and pemvidutide, are also under investigation, although longer-term phase 3 data and comparative studies are needed to define their safety, durability, body-composition effects, and clinical positioning. In parallel, oral small-molecule GLP-1 receptor agonists such as orforglipron may broaden treatment options by addressing route of administration and access-related barriers, although they should be considered separately from dual- or multi-receptor agonist strategies [45].

These findings suggest that multi-receptor agonism may further increase the magnitude of pharmacologically induced weight loss, although larger and longer-term studies are needed to define durability, safety, body-composition effects, functional outcomes, and clinical applicability.

### 3.3. Unresolved Challenges Beyond Body Weight: Body Composition, Tolerability and Durability of Response

While novel incretin-based pharmacotherapies are highly effective in reducing body weight, several clinically relevant challenges remain beyond the magnitude of weight loss alone. As pharmacologically induced weight loss becomes increasingly substantial, greater attention should be paid to the quality, functional implications, tolerability, real-world persistence, and durability of treatment response.

One of the main concerns is that rapid weight loss may lead to a decrease in fat-free mass, lean mass (LM), and skeletal muscle mass (SMM). In the context of intentional weight loss, a variable proportion of overall weight loss can be due to LM loss, and recent analyses suggest that lean mass may account for approximately 25–39% of weight lost during incretin-based pharmacotherapy [13,46]. This range should not be interpreted as a single pooled estimate, as the available studies differ in treatment, duration, magnitude of weight loss, diabetes status, age, sex distribution, baseline adiposity, and body-composition assessment. Importantly, dual-energy X-ray absorptiometry-derived lean mass does not correspond exclusively to skeletal muscle, but also includes organs, connective tissue, body water, and other non-fat components. Therefore, reductions in lean mass should not automatically be interpreted as pure skeletal muscle loss.

Loss of LM and SMM has clinical significance, especially in older people, in those with low baseline muscle mass, and sarcopenic obese patients. Even moderate amounts of skeletal muscle loss in these populations may negatively affect strength, resting energy expenditure, physical function, frailty risk, and weight maintenance, particularly in vulnerable populations [47,48]. Furthermore, the evidence is not consistently evaluated in terms of muscle strength, physical performance, cardiorespiratory fitness, or functional capacity, and it is unclear how changes in LM translate to clinically meaningful functional impairment [49,50]. Bone health may also require attention during substantial weight loss, particularly in older adults and individuals at increased fracture risk [51,52,53,54].

Gastrointestinal adverse events, including nausea, vomiting, diarrhea, and constipation, may contribute to dose reduction, treatment interruption, or discontinuation [28,38]. Persistence in real-world settings is generally lower than in pivotal clinical trials and may be influenced by tolerability, cost, access, drug availability, treatment expectations, and the availability of multidisciplinary support [55].

Finally, the durability of weight loss after medication withdrawal remains a key unresolved issue. Participants in the STEP 1 extension regained a substantial proportion of the weight previously lost after semaglutide discontinuation, while SURMOUNT-4 showed weight regain among participants switched from tirzepatide to placebo and further weight loss among those continuing treatment [56,57].

This pattern underscores that a major limitation of incretin-based pharmacotherapy is not short-term efficacy, but the challenge of translating pharmacologically induced weight loss into durable metabolic, functional, and behavioral improvements. This limitation provides the rationale for integrating PA, nutritional strategies, behavioral support, and long-term monitoring into obesity pharmacotherapy. Table 1 summarizes the principal pharmacological approaches of current and emerging anti-obesity therapies and their implications for lifestyle co-therapy.

## 4. Physical Activity as a Co-Therapy During Pharmacological Weight Loss

Although incretin-based medications can induce clinically meaningful reductions in body weight, pharmacological weight loss does not automatically guarantee optimal improvements in body composition, physical function, bone health, or long-term weight maintenance [58]. Therefore, PA should not be considered merely a tool to increase energy expenditure, but rather a therapeutic component that may improve cardiometabolic health, preserve musculoskeletal function, and support long-term weight-management strategies [59].

This concept is consistent with recent cardiometabolic and lifestyle medicine recommendations emphasizing that PA remains a core component of obesity care even in the era of highly effective anti-obesity medications [60]. Similarly, recent narrative reviews on lifestyle medicine and incretin-based medications highlight that structured PA may help optimize weight-loss quality, treatment tolerability, lean-mass preservation, functional capacity, and weight-loss maintenance [61,62,63]. However, the strength of the available evidence varies considerably. Direct randomized evidence evaluating structured exercise during incretin-based pharmacotherapy remains limited and is currently based mainly on studies involving liraglutide rather than newer agents such as semaglutide or tirzepatide. Much of the current rationale is therefore derived from exercise studies conducted during diet-induced weight loss, obesity treatment without pharmacotherapy, or mechanistic investigations. These findings are clinically important because they are informative but should not be interpreted as definitive evidence of the effects of structured exercise during treatment with newer incretin-based agents.

Weight loss, whether induced by CR, bariatric surgery, or pharmacotherapy, may be accompanied by reductions in fat-free mass and bone mineral density [26]. These changes may become clinically relevant, especially in older adults, individuals with sarcopenic obesity, patients with low baseline fitness, and those with obesity-related functional limitations. Exercise training may help attenuate these unfavorable adaptations by stimulating muscle protein synthesis, improving neuromuscular function, enhancing cardiorespiratory fitness, supporting bone health, and improving mobility and functional reserve [51,63].

Evidence from diet-induced weight-loss interventions suggests that exercise, particularly combined aerobic and resistance training, may improve physical function and attenuate lean-mass and bone loss. However, these findings provide indirect support and have not been confirmed during treatment with newer incretin-based agents [12,51].

Despite this strong rationale, direct evidence evaluating structured exercise programs specifically during incretin-based pharmacotherapy remains limited. Pivotal trials such as STEP 1 and SURMOUNT-1 included lifestyle counseling, recommendations for CR, and general advice to increase PA [26,36], but did not test supervised exercise as an independent therapeutic component. In this context, SURMOUNT-3 provides particularly relevant evidence for an integrated treatment model. In this phase 3 trial, adults with overweight or obesity without diabetes first underwent a 12-week intensive lifestyle intervention and were randomized only if they achieved at least 5% weight reduction. Participants then received once-weekly tirzepatide, at the maximum tolerated dose of 10 or 15 mg, or placebo for 72 weeks. Tirzepatide produced a further mean body-weight reduction of 18.4%, whereas placebo was associated with a 2.5% weight increase from randomization. In addition, 87.5% of participants receiving tirzepatide achieved at least an additional 5% weight reduction compared with 16.5% receiving placebo. Although SURMOUNT-3 did not isolate exercise as an independent component, it supports a sequential and integrated care model in which intensive lifestyle intervention and pharmacotherapy are combined rather than considered alternative strategies [39].

### 4.1. Aerobic Exercise and Cardiometabolic Adaptations

Aerobic exercise complements pharmacological weight loss by improving cardiorespiratory fitness, insulin sensitivity, vascular function, and skeletal muscle oxidative capacity [64,65,66]. These benefits are clinically relevant because cardiorespiratory fitness predicts morbidity and mortality independently of body weight [67,68].

However, most evidence supporting aerobic exercise during weight reduction is indirect. Lifestyle-based trials have shown that aerobic training improves peak oxygen uptake during CR, whereas combined aerobic and resistance training provides additional benefits for strength and lean mass preservation [12]. These findings are relevant for patients treated with anti-obesity pharmacotherapies because large pharmacological weight reductions may improve relative fitness but may not necessarily improve absolute aerobic capacity or functional reserve unless exercise training is included.

The randomized trial by Lundgren et al. represents one of the most relevant examples of structured exercise combined with incretin-based pharmacotherapy [69]. After an initial low-calorie diet-induced weight-loss phase, adults with obesity were assigned to exercise, liraglutide, combined exercise plus liraglutide, or placebo for 52 weeks. The combined intervention produced greater reductions in body weight and body-fat percentage than either intervention alone and was associated with favorable effects on insulin sensitivity and cardiorespiratory fitness. Nevertheless, this evidence relates specifically to liraglutide and should not be directly generalized to semaglutide, tirzepatide, or newer multi-receptor agonists.

Additional physiological evidence suggests that exercise may interact with incretin-related metabolic pathways. Endurance training has been reported to improve β-cell sensitivity to GLP-1 and glucose tolerance in overweight women, while an acute treadmill-walking session increased β-cell sensitivity to GLP-1 in individuals with type 2 diabetes [70,71]. These studies support biological plausibility but did not evaluate exercise in combination with anti-obesity pharmacotherapy and therefore cannot establish an additive clinical effect.

**Figure 2:**
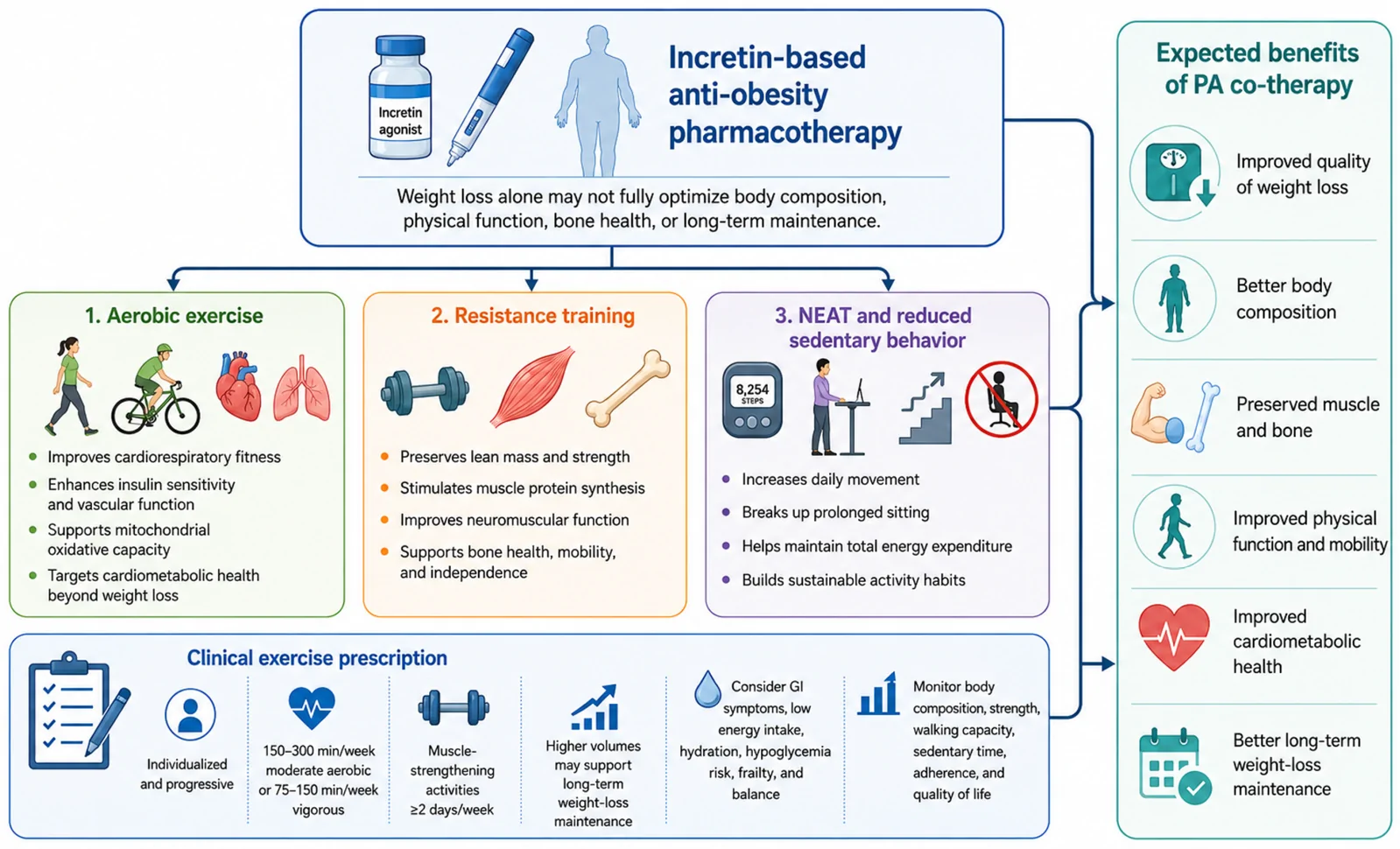
Physical activity as co-therapy during pharmacological weight loss. Aerobic exercise, resistance training, NEAT, and reduced sedentary behavior may complement anti-obesity pharmacotherapy by supporting cardiometabolic health, muscle function, daily movement, exercise safety, and long-term weight-loss maintenance. Exercise should be prescribed not only to support energy expenditure, but also to preserve cardiorespiratory fitness, muscle function, and daily movement during pharmacological weight loss.

However, direct evidence on structured aerobic exercise during treatment with newer incretin-based agents remains limited, as most pivotal semaglutide and tirzepatide trials incorporated lifestyle counseling rather than supervised aerobic training and did not isolate the independent contribution of exercise [26,36]. Consequently, findings derived from liraglutide-based interventions or non-pharmacological weight-loss studies should not be directly generalized to semaglutide, tirzepatide, or newer multi-receptor agonists. Future studies should assess cardiorespiratory fitness, functional capacity, fatigue, and quality of life in addition to body weight. The main cardiometabolic and fitness benefits of aerobic exercise as a co-therapy are summarized in Figure 2.

### 4.2. Resistance Training and Muscle Preservation

Resistance training directly targets SMM, strength, neuromuscular function, and protects bone health [72]. As discussed in Section 3.3, reductions in DXA-derived lean mass should not be automatically interpreted as pure skeletal muscle loss, because lean mass also includes body water, organs, connective tissue, and other non-fat components [73,74].

The physiological rationale for resistance training during pharmacological weight loss is strong. Progressive resistance exercise stimulates muscle protein synthesis, counteracting some of the catabolic effects associated with CR and rapid weight reduction [75,76]. In older adults with obesity undergoing weight-loss therapy, combined aerobic and resistance training improved muscle protein synthesis and preserved myocellular quality despite CR [77]. These findings provide a relevant mechanistic and clinical rationale, but they were not obtained during incretin-based pharmacotherapy.

Most available evidence on body-composition preservation is therefore indirect. In a retrospective cohort study including 304 adults undergoing a hypocaloric diet, Lahav et al. compared resistance training, aerobic exercise, and no exercise during weight reduction [72]. Although total weight loss was similar across groups, resistance training produced the most favorable body-composition profile, with greater fat-mass reduction and preservation or increase in fat-free mass. Similarly, in a randomized trial comparing aerobic training, resistance training, and combined aerobic plus resistance training during diet-induced weight loss in older adults with obesity, resistance and combined training were more effective than aerobic training alone in limiting lean mass loss, while combined training produced the most favorable overall effects on physical function, strength, and cardiorespiratory fitness [12]. These studies support the important role of resistance training as a core component of obesity care, although their findings cannot be assumed to apply directly to semaglutide, tirzepatide, or other newer agents.

More directly, Locatelli et al. specifically proposed that tailored resistance exercise should be recommended alongside incretin-based weight-loss pharmacotherapy to optimize body-composition changes by preserving lean mass while promoting fat loss [78].

To date, direct clinical evidence remains limited and is derived mainly from studies involving liraglutide. In the randomized trial by Lundgren et al., exercise alone increased lean mass, liraglutide alone maintained lean mass, and combined exercise plus liraglutide produced a modest increase in lean mass, although between-group differences were not statistically significant. Importantly, the combination of exercise and liraglutide produced greater fat-mass reduction than either intervention alone, suggesting that structured exercise may improve the quality of pharmacologically supported weight-loss maintenance by favoring fat loss while limiting unfavorable changes in lean tissue [69].

Resistance and combined exercise may also help protect bone health during pharmacological weight loss. A secondary analysis of the Lundgren trial showed that liraglutide alone reduced BMD at the hip and spine compared with placebo or exercise alone, whereas combined exercise and liraglutide preserved hip and spine BMD despite larger weight loss [79]. Although clinically relevant, this result derives from a single intervention context and requires confirmation with semaglutide, tirzepatide, and newer multi-receptor agonists.

Overall, resistance training represents a clinically relevant component of obesity care because of its established effects on strength, mobility, balance, bone health, and functional independence. However, direct evidence regarding its effects during treatment with semaglutide, tirzepatide, or newer multi-receptor agonists remains insufficient. Findings derived from diet-induced weight loss and liraglutide-based interventions should therefore not be generalized to newer and more potent agents without appropriate caution. Future trials should include structured resistance protocols and assess skeletal muscle mass, muscle strength, physical performance, bone health, adherence, and patient-centered functional outcomes. The protective effects on lean mass and physical function are outlined in Figure 2.

### 4.3. NEAT and Sedentary Behavior

Non-exercise activity thermogenesis refers to the daily energy expenditure attributable to spontaneous physical activities (e.g., walking, standing, occupational activities, fidgeting) [80,81].

Observational evidence indicates that individuals with obesity may spend more time seated than lean individuals, although these findings were obtained outside pharmacological treatment [82].

During pharmacological weight loss, NEAT may become clinically relevant for two main reasons. First, as body weight decreases, the energy cost of movement also declines; therefore, maintaining the same amount of daily activity may result in lower total energy expenditure over time. Second, pharmacologically induced weight loss may occur without parallel improvements in habitual movement patterns. Therefore, daily activity should be intentionally preserved or progressively increased during treatment, rather than assumed to improve automatically as body weight decreases [83].

Sedentary behavior should be considered as a related but distinct therapeutic target. Prolonged sedentary time is associated with increased risk of adverse health outcomes, including cardiovascular disease, diabetes, all-cause mortality, and hospitalization [84]. Accordingly, the WHO 2020 guidelines recommend that adults reduce sedentary behavior and replace it with PA of any intensity, in addition to achieving regular aerobic and muscle-strengthening activity [85]. These recommendations are relevant to obesity care in general, but their specific effects during pharmacological treatment have not been adequately tested.

In practical terms, NEAT and sedentary behavior should be addressed alongside structured exercise prescription. Strategies such as increasing daily step count, interrupting prolonged sitting, walking after meals, standing during routine tasks, using stairs when feasible, and incorporating short bouts of light-intensity activity throughout the day may help patients increase total daily movement. These strategies should not be considered substitutes for aerobic or resistance training, but complementary tools to increase total daily movement and improve adherence, particularly in individuals with low baseline fitness, joint pain, severe obesity, fatigue, or psychological barriers to formal exercise.

At present, evidence directly evaluating NEAT or sedentary behavior during treatment with GLP-1 receptor agonists or dual GIP/GLP-1 receptor agonists remains limited. The contribution of NEAT and reduced sedentary behavior to daily movement and overall outcomes is shown in Figure 2.

### 4.4. Clinical Considerations for Exercise Prescription During Anti-Obesity Pharmacotherapy

Exercise prescription during anti-obesity pharmacotherapy should be individualized, progressive, and clinically realistic. Current PA guidance supports the integration of aerobic activity, muscle-strengthening exercise, and reductions in sedentary time as part of comprehensive obesity care. The 2026 American Heart Association Scientific Statement further emphasizes that PA can complement diet-induced energy deficit, pharmacotherapy, and bariatric surgery by supporting weight-loss maintenance and improving cardiometabolic outcomes, even when its independent effect on weight loss is modest [60]. General guidelines recommend at least 150–300 min per week of moderate-intensity aerobic activity, or 75–150 min per week of vigorous-intensity aerobic activity, together with muscle-strengthening activities involving major muscle groups on at least two days per week [85]. For long-term weight-loss maintenance, higher levels of PA are often required. The American College of Sports Medicine position stand reported that 150–250 min per week of moderate-intensity PA may prevent weight gain and produce only modest weight loss, whereas greater amounts, often exceeding 250 min per week, are associated with clinically significant weight loss and better prevention of weight regain [86]. Consistently, higher levels of PA have been associated with better long-term weight-loss maintenance [87]. However, these recommendations are largely derived from evidence in the general population and from lifestyle-based obesity interventions rather than from trials specifically evaluating structured exercise during treatment with newer incretin-based agents.

In clinical practice, exercise should be introduced gradually to reduce injury risk and accommodate joint pain, low fitness, dyspnea, fatigue, or balance impairment. Aerobic activity should be adapted to individual tolerance, while resistance training may progressively reach two to three sessions per week when feasible [88,89]. Alongside structured sessions, clinicians should provide actionable strategies to preserve daily movement, such as increasing step counts, interrupting prolonged sitting, and incorporating short bouts of light-intensity activity throughout the day [90,91].

Wearable activity trackers and smartwatches may support these goals by enabling self-monitoring of daily step count, activity intensity, sedentary time, and heart rate, while also providing reminders and individualized activity targets. These tools may facilitate feedback, reinforce gradual progression, and help clinicians monitor adherence outside supervised exercise sessions. However, they should be considered adjuncts to individualized counseling and exercise prescription, with attention to device accuracy, patient preferences, and accessibility. The role of wearable-based self-monitoring in long-term weight-loss maintenance is further discussed in Section 8.2.

In patients treated with incretin-based agents, exercise prescription must consider medication-specific challenges. During treatment initiation and dose escalation, gastrointestinal symptoms (nausea, vomiting, diarrhea) and reduced energy intake can affect exercise tolerance. Consequently, exercise intensity, timing, and modality may need temporary adjustment [92]. If reduced oral intake or gastrointestinal adverse effects increase dehydration risk, individualized hydration strategies are essential before, during, and after exercise. Furthermore, activities requiring balance, aquatic settings, or unsupervised vigorous exercise warrant additional caution if dizziness, marked fatigue, or medication-related symptoms impair patient safety [93].

Safety considerations are particularly important in patients with diabetes, older adults, individuals with frailty risk, and patients taking additional medications that may influence blood pressure, glucose levels, alertness, or heat tolerance. In patients with diabetes, particularly those receiving insulin or insulin secretagogues, exercise-related hypoglycemia should also be carefully monitored [94]. In older adults, postmenopausal women, individuals with sarcopenic obesity, and patients at risk of frailty, combining resistance training with balance and mobility exercises is critical to preserve functional independence and reduce fall risk [95].

Monitoring should extend beyond body weight and include body composition, muscle strength, walking capacity, fatigue, sedentary time, adherence, safety, and quality of life. When feasible, body-composition assessment and simple functional tests, such as handgrip strength, chair-stand performance, and gait speed, may provide clinically useful information [96,97].

Finally, exercise counseling should be patient-centered and non-stigmatizing. Many individuals with obesity have experienced weight stigma in healthcare and exercise settings, which may reduce exercise self-efficacy and promote avoidance of PA. Framing exercise around health, function, energy, mobility, and independence rather than appearance or body weight alone may improve adherence and long-term engagement [98].

Future trials should move beyond generic lifestyle advice and test structured exercise protocols during newer multi-receptor agonist treatments to define the independent contribution of exercise during pharmacological obesity treatment. These studies could clarify the independent and combined effects of aerobic training, resistance training, balance/mobility exercise, NEAT promotion, and sedentary-time reduction on body composition, physical function, cardiometabolic health, treatment adherence, and long-term weight-loss maintenance. The core principles for a safe clinical exercise prescription are summarized in the lower panel of Figure 2.

## 5. Nutritional Strategies Supporting Pharmacological Weight Loss

Pharmacologically induced appetite suppression creates specific nutritional challenges during obesity treatment. Incretin-based therapies reduce hunger, increase satiety, delay gastric emptying, and decrease total energy intake through central and peripheral mechanisms [99]. However, a lower quantity of food does not necessarily translate into a higher-quality diet. When nutritional care is not actively integrated into treatment, patients may lose weight while consuming insufficient protein, fiber, fluids, and micronutrients, particularly during treatment initiation, dose escalation, or periods of gastrointestinal intolerance [100,101].

Nutritional care should therefore support diet quality, lean-tissue preservation, gastrointestinal tolerability, and sustainable eating patterns [102].

However, direct trials comparing specific dietary interventions during newer incretin-based pharmacotherapy remain limited. Current recommendations are largely derived from established nutritional principles and non-pharmacological weight-loss studies and should therefore be interpreted as clinically informed strategies rather than pharmacotherapy-specific protocols [103].

**Figure 3:**
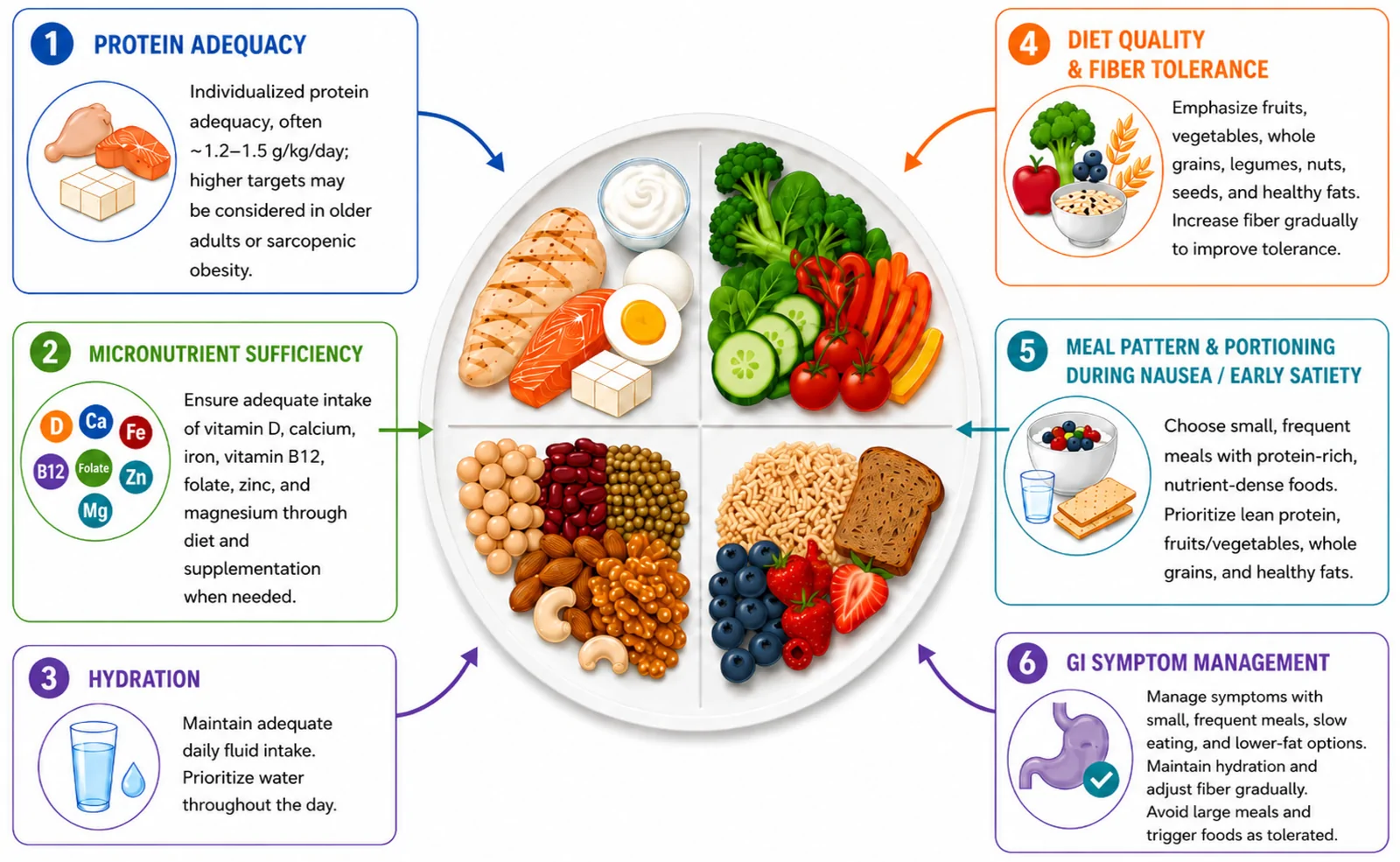
Nutritional priorities during GLP-1/GIP-based weight loss. During pharmacological appetite suppression, nutritional care should support protein adequacy, micronutrient sufficiency, hydration, gastrointestinal tolerability, diet quality, and lean-tissue preservation.

The following sections address diet quality during appetite suppression, protein intake and lean-mass preservation, micronutrient adequacy, hydration and gastrointestinal management, and individualized nutritional monitoring. The main nutritional priorities during GLP-1/GIP-based weight loss are summarized in Figure 3.

### 5.1. Diet Quality During Appetite Suppression

Incretin-based therapies can substantially reduce appetite and meal size, but this effect does not automatically improve dietary quality [104]. Some patients may simply eat smaller portions of foods that are easy to tolerate, convenient, or highly palatable, rather than foods that are nutritionally adequate. This may result in a diet that is lower in calories but also lower in protein, fiber, vitamins, minerals, and dietary variety [105,106].

Diet quality is particularly important because the metabolic benefits of weight loss may be influenced by the type of foods consumed during treatment. Dietary patterns emphasizing vegetables, fruits, legumes, whole grains, lean protein sources, unsaturated fats, and minimally processed foods have been associated with better cardiometabolic profiles than dietary patterns rich in ultra-processed foods, refined carbohydrates, added sugars, and saturated fats [107]. A meta-analysis of numerous clinical trials revealed that in patients with type-2 diabetes, Mediterranean diets improved weight loss compared to control diets [108]. It is to notice that this evidence derives mainly from general obesity and diabetes populations rather than from trials specifically conducted during incretin-based pharmacotherapy. Direct comparisons of defined dietary patterns in patients receiving semaglutide, tirzepatide, or newer multi-receptor agonists remain limited.

No single dietary pattern can therefore be considered universally optimal during pharmacological weight loss; adherence, tolerability, cultural preferences, comorbidities, and patient goals should guide individualized dietary planning [109].

Among the most studied patterns, the Mediterranean-style diet is consistently associated with improved cardiometabolic outcomes and is characterized by a high intake of vegetables, fruits, legumes, whole grains, unsaturated fats, and lean protein sources [110,111]. Similarly, Dietary Approaches to Stop Hypertension (DASH)-style dietary patterns have been linked to improvements in blood pressure control and overall cardiometabolic risk reduction, reflecting the importance of nutrient-dense food selection rather than macronutrient restriction alone [112]. Low-fat and low-carbohydrate dietary approaches may both be effective for weight management; however, their success is primarily determined by adherence, energy intake, food quality, and individual patient preferences rather than inherent superiority of one macronutrient composition over another [113,114]. Other dietary patterns may also be considered. The healthy Nordic diet, which emphasizes whole grains, vegetables, berries, fish, and unsaturated fats, may provide modest benefits for weight control and cardiometabolic health [113]. Ketogenic diets may promote short-term weight and fat-mass reduction, but concerns remain regarding long-term adherence, dietary variety, fiber and micronutrient adequacy, and gastrointestinal tolerability [114,115]. These issues may be particularly relevant during incretin-based pharmacotherapy, and both approaches should be individualized, as direct evidence in this treatment setting remains limited. Plant-based or vegetarian dietary patterns may also be appropriate when carefully planned, but during periods of reduced food intake they require particular attention to protein adequacy and micronutrient sufficiency, including vitamin B12, iron, zinc, calcium, and vitamin D [115]. In addition, higher-protein dietary strategies may be considered briefly within this context, although their detailed role in lean mass preservation is addressed separately [76].

Overall, dietary pattern selection during incretin therapy should be individualized and guided by nutrient density, cardiometabolic profile, gastrointestinal tolerance, micronutrient risk, cultural preferences, and long-term sustainability rather than a single prescriptive diet model. Importantly, even otherwise healthy dietary patterns may require modification during treatment initiation or dose escalation, as gastrointestinal side effects such as nausea, early satiety, and bloating may limit tolerance of high-fiber, high-fat, or highly fermentable foods in some patients [116].

While GLP-1 receptor agonists and other incretin-based therapies have been proven to enhance blood glucose control, the quality of carbohydrates in the diet still needs to be taken into account from a clinical standpoint [117]. Refined carbohydrates and added sugars can still cause postprandial hyperglycemia, particularly in people with obesity, insulin resistance or type 2 diabetes [118]. Low-glycemic-index and low-glycemic-load dietary patterns may support glycemic control and improve satiety, while higher-fiber foods may slow carbohydrate absorption, increase fullness, and promote the production of short-chain fatty acids through gut microbiota fermentation [119,120]. There is also new evidence that fermentable dietary fibers and short-chain fatty acids (SCFAs) may promote endogenous GLP-1 secretion, and that this may offer a new complementary approach to incretin-based therapy in obesity and metabolic disease [121,122].

Meal composition also affects gastrointestinal tolerability. Large meals and high-fat meals may aggravate nausea, bloating, reflux, and early satiety because dietary fat slows gastric emptying and may amplify drug-related gastrointestinal symptoms [123]. During treatment initiation or dose escalation, smaller meals, slower eating, reduced meal fat load, and avoidance of very large portions may improve tolerability and adherence [124].

Lastly, dietary strategies for weight management should not be limited to calorie deficit. Sustainability requires a personalized approach, considering lifestyles, cultural preferences, and behavioral patterns [125]. Strict diets can cause psychological stress and can lead to disordered eating habits, while more flexible and balanced eating patterns are more likely to help people stick to their diets in the long term [126].

### 5.2. Protein Intake and Lean Mass Preservation

During pharmacological weight loss, dietary protein has clinical importance. Incretin-based therapies are known to lower appetite and overall calorie consumption. Consequently, patients may also have inadequate protein intake to support muscle growth and maintenance of lean body mass [101,127].

The effect of dietary protein on muscle is not only dependent on the total daily protein intake, but also on the time of protein consumption, the source, quality, and the metabolic state of the patient. High-quality protein sources like whey protein, eggs, meat, and dairy are excellent for stimulating muscle protein synthesis [128,129,130]. Protein may be well tolerated if taken frequently in small amounts rather than taken in larger amounts at once [131]. Patients taking incretin-based therapy may find it challenging to achieve a sufficient protein intake at each meal due to decreased hunger and meal size. So, planned protein consumption is essential to ensure protein is anabolically stimulated several times during the day [16,127].

The *2025–2030 Dietary Guidelines for Americans*, released in 2026, recommend a protein intake of 1.2–1.6 g/kg/day, whereas the traditional intake of 0.8 g/kg/day represents the minimum recommended allowance for generally healthy adults rather than an optimal target during intentional weight loss [132]. This updated range is consistent with evidence indicating greater protein requirements during energy restriction. A systematic review and meta-analysis of available evidence published by Kokura et al. in 2024 [133], which included 47 randomized controlled trials and 3218 adults with overweight or obesity, revealed that higher protein consumption more than 1.3 g/kg/day was linked to maintaining or improving muscle mass during energy deficit. However, protein deficiencies less than 1.0 g/kg/day were associated with an increased risk of muscle mass loss. The results provide evidence for the clinical guidelines of 1.2–1.5 g/kg/day during weight loss, and of 1.5–2.0 g/kg/day in older people or patients with sarcopenic obesity [133,134]. However, these ranges have not been specifically validated during newer incretin-based pharmacotherapy and should be individualized according to kidney function, comorbidities, baseline intake, body composition, dietary preferences, and tolerance.

The anabolic response to dietary protein depends on total daily intake, protein quality, and its distribution across meals, as well as individual factors such as age, baseline lean mass, and metabolic status. Evidence from weight-loss and resistance training studies indicates that higher protein intake enhances lean mass retention, particularly when combined with structured exercise interventions [135,136]. However, protein supplementation alone is insufficient to fully prevent lean mass loss in the absence of exercise, and its effectiveness varies depending on total intake, distribution, and individual metabolic characteristics. Clinical studies suggest that combined protein and resistance exercise interventions produce more consistent preservation of lean mass compared with either strategy alone [137,138]. Anyway, direct randomized evidence establishing the optimal protein intake during semaglutide, tirzepatide, or newer multi-receptor agonist therapy remains limited.

Protein is also an important factor that affects resting energy expenditure, which is strongly related to fat-free mass, notably skeletal muscle mass. Therefore, the loss of lean tissue during weight reduction contributes to an expected decrease in energy expenditure [139,140]. This expected decrease should be distinguished from adaptive thermogenesis, which refers to a reduction in energy expenditure beyond that predicted by changes in body weight and body composition [141]. These physiological changes may contribute to the difficulty of maintaining weight loss after treatment discontinuation [142]. Resistance training (as discussed in Section 4.2), combined with adequate protein intake, can help to preserve muscle mass by maintaining metabolically active lean tissue and may contribute to a higher resting energy expenditure per unit of body mass [143].

The tolerability of the treatment may also influence protein intake. Gastrointestinal symptoms and early satiety during treatment initiation or dose escalation may make it difficult for some patients to achieve their protein targets [127,144,145]. Protein intake should therefore be individualized according to symptoms and tolerance, using smaller portions and well-tolerated protein-rich foods when necessary. Special attention should be paid to older adults since they already have age-related anabolic resistance and are more susceptible to lean tissue loss in calorie-restricted diets [146,147]. Furthermore, guidelines suggest that, in older patients, consuming 25–30 g of high-quality protein after each meal may stimulate postprandial anabolic response [146,148]. In these groups, even a small loss of lean body mass can lead to a loss of strength, PA, and functional independence [61]. Therefore, protein optimization should be a core component of pharmacological obesity treatment in older adults rather than an optional intervention.

Before protein loss and/or functional impairment is apparent, clinicians should evaluate a patient’s normal food intake, eating habits, gastrointestinal tolerance, and cultural food choices [16,127]. There are validated nutrition-risk screening tools, such as the Mini Nutritional Assessment for older adults and the Malnutrition Universal Screening Tool for adults, that can be used to identify the risk for poor protein intake and to determine the level of dietetic support required [149,150]. Using these assessment tools may help to design the nutritional plan to reduce the risk of loss of lean body mass during weight loss treatment.

### 5.3. Micronutrient Adequacy, Hydration, and Gastrointestinal Management

Reduced food intake during incretin-based pharmacotherapy may increase the risk of micronutrient inadequacy, particularly when dietary variety decreases or gastrointestinal symptoms limit intake [144]. However, direct evidence quantifying the prevalence and clinical relevance of specific micronutrient deficiencies during treatment with newer incretin-based agents remains limited. Current concerns are therefore based largely on reduced dietary exposure, individual risk factors, and evidence from CR and other weight-loss settings.

Vitamin D and calcium are especially important because weight loss can reduce mechanical loading on bone, have an impact on bone turnover, and contribute to concerns for bone health, especially in older adults and postmenopausal women [151]. Iron, folate, and vitamin B12 may also be deficient if patients do not tolerate or consume enough red meat, legumes, or fortified foods. Magnesium and zinc are also relevant for glucose metabolism, immune function, oxidative stress, and muscle function, and may be insufficient in diets dominated by ultra-processed foods or limited dietary variety [152]. Currently, few specific studies have been conducted in the setting of incretin-based pharmacotherapy; however, evidence from the CR and bariatric surgery literature suggests that monitoring, and potentially supplementation, should be carried out early if micronutrient deficiencies are suspected [153].

Reduced food intake, vomiting, and diarrhea may compromise hydration and increase the risk of postural symptoms, particularly during dose escalation and in older adults. Hydration strategies should therefore be individualized according to intake, symptoms, comorbidities, and clinical risk [154]. Therefore, hydration strategies should be individualized and discussed early, especially during dose escalation or periods of gastrointestinal symptoms.

Gastrointestinal management should be integrated into nutritional care because tolerability strongly influences adherence and nutrient adequacy. Nausea, vomiting, constipation, diarrhea, reflux, bloating, and early satiety are among the most common symptoms reported with GLP-1 receptor agonists and dual incretin therapies [132]. Practical strategies may include smaller meals, slower eating, reduced meal fat load, avoidance of very large meals, adequate hydration, gradual fiber adjustment, and temporary modification of foods that worsen bloating or nausea [115,116]. For constipation, fiber and fluids may help, but fiber should be titrated gradually and matched with adequate hydration. For diarrhea or marked bloating, temporary reduction in very high-fat foods, sugar alcohols, and selected fermentable foods may be useful, followed by reintroduction according to tolerance.

The key point is that gastrointestinal dietary management should not be framed only as symptom control. If symptoms reduce protein, fluid, or micronutrient intake, they may indirectly affect lean-mass preservation, exercise tolerance, adherence, and overall treatment quality. Therefore, gastrointestinal management is part of nutritional optimization, not a secondary issue [155].

### 5.4. Nutritional Monitoring and Individualized Care

Nutritional monitoring should be integrated from the initiation of anti-obesity pharmacotherapy, rather than introduced only after clinically evident nutritional or functional compromise has developed. Because incretin-based therapies reduce appetite, meal size, and total energy intake, patients may progressively reduce dietary variety, protein intake, fluid intake, and micronutrient exposure, particularly during dose escalation or periods of gastrointestinal intolerance. Therefore, monitoring should aim to detect early signs of nutritional risk and support the quality, tolerability, and functional outcomes of weight loss [155]. In addition, closer nutritional monitoring may be particularly relevant in individuals following restrictive or low-variety dietary patterns during incretin-based pharmacotherapy [156]. Although such dietary patterns can be nutritionally adequate when well planned, appetite suppression and early satiety induced by GLP-1 receptor agonists and dual incretin therapies may further reduce dietary flexibility and overall intake [26]. Consequently, there is a potential risk of inadequate protein consumption, reduced micronutrient density, limited fiber tolerance, and suboptimal hydration if dietary intake is not actively monitored and individualized. Evidence from nutritional and incretin physiology literature also indicates that dietary composition can significantly influence gut hormone responses, including endogenous GLP-1 secretion, highlighting the importance of diet quality and composition during pharmacological treatment [157]. Furthermore, recent narrative reviews emphasize that real-world incretin-based therapy outcomes are strongly influenced by dietary adequacy, gastrointestinal tolerability, and nutritional support strategies, supporting the need for structured monitoring in high-risk dietary patterns [158,159,160]. Therefore, dietary pattern considerations should be integrated into routine follow-up as part of individualized nutritional assessment, ensuring that pharmacologically reduced food intake does not compromise nutritional adequacy, functional outcomes, or long-term treatment sustainability.

Baseline assessment should include habitual dietary intake, meal pattern, protein intake, fluid intake, gastrointestinal history, food intolerances, previous weight-loss attempts, comorbidities, medications, physical activity level, and risk factors for malnutrition or micronutrient inadequacy. Closer monitoring is warranted in fragile patients such as older adults, postmenopausal women, and individuals with sarcopenic obesity.

Laboratory monitoring should be tailored to individual clinical risk. There is currently insufficient evidence to recommend routine indiscriminate micronutrient panels for all patients; however, targeted assessment may be warranted in those with poor dietary intake, persistent gastrointestinal symptoms, older age, rapid or large weight loss, restrictive diets, suspected malnutrition, or pre-existing nutritional risk. Depending on clinical context, laboratory evaluation may include vitamin D and calcium-related parameters, iron status, folate, vitamin B12, renal function, electrolytes, and other markers guided by symptoms, comorbidities, dietary pattern, and local practice. This cautious approach is consistent with evidence showing that GLP-1 and dual GIP/GLP-1 receptor agonists reduce total energy intake, while data on diet composition and micronutrient adequacy remain limited [161].

Supplementation should be targeted rather than routine. Vitamin, mineral, protein, or fiber supplements may be considered, where appropriate and tolerated, if dietary intake is inadequate, biochemical deficiency is documented or strongly suspected, gastrointestinal symptoms restrict food variety, or the patient cannot achieve protein and micronutrient requirements from dietary sources [155]. Supplements should complement, not replace, a structured dietary pattern based on nutrient-dense foods, adequate protein distribution, hydration, and individualized gastrointestinal management.

Nutritional care should also be planned in conjunction with physical activity prescription. Adequate protein intake provides the substrate needed to support lean-tissue preservation, whereas resistance training provides the anabolic stimulus required to maintain strength, muscle quality, and functional capacity during weight loss [11,12,76]. Likewise, when patients increase aerobic or resistance training, particularly during periods of dose escalation, reduced oral intake, or gastrointestinal symptoms, hydration and carbohydrate availability may need to be considered according to exercise volume, symptoms, and metabolic risk [98,162]. Therefore, nutrition and physical activity should be addressed as interacting components of the same co-therapeutic plan during anti-obesity pharmacotherapy [98,155].

Dietetic support may be especially beneficial in individuals with persistent gastrointestinal symptoms, low protein intake, low dietary diversity, micronutrient inadequacy or risk, weight cycling, disordered eating concerns, or multiple cardiometabolic comorbidities. Qualified nutrition professionals may provide medical nutrition therapy to support medication adherence, adverse-effect management, adequate nutrient intake, and the development of sustainable lifestyle behaviors during and after pharmacological weight loss [163].

Overall, individualized nutritional monitoring provides the clinical link between pharmacological appetite suppression and high-quality weight loss. Its purpose is not to intensify CR, but to ensure that reduced food intake remains nutritionally adequate, gastrointestinal symptoms are managed, lean tissue and physical function are supported, and sustainable dietary habits are developed to promote long-term weight-loss maintenance. Key PA and nutritional priorities are summarized in Table 2.

## 6. Neurobehavioral and Lifestyle Interactions in Pharmacological Weight Loss

**Figure 4:**
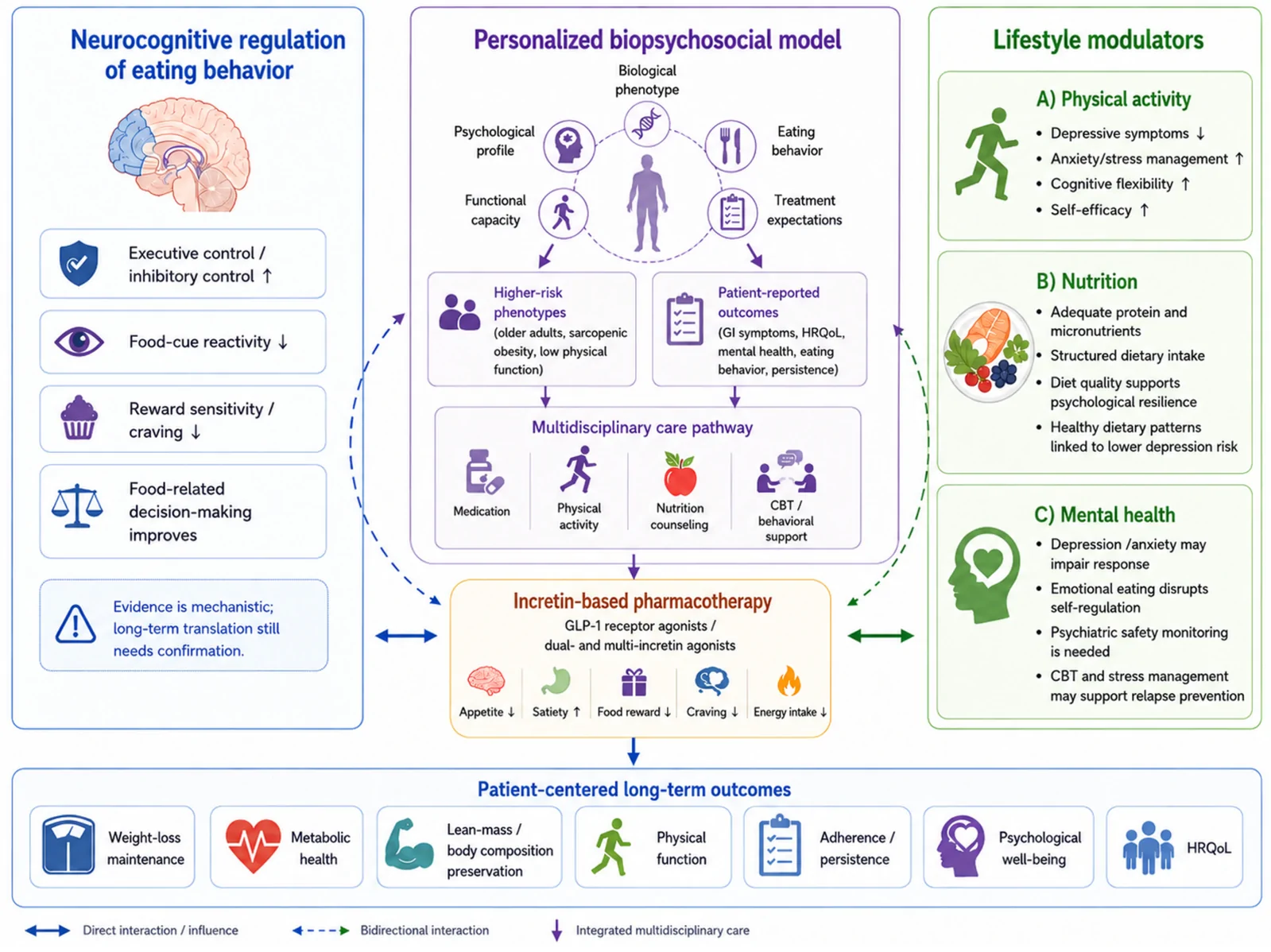
Neurobehavioral and biopsychosocial factors influencing treatment response. Long-term treatment response depends not only on pharmacological appetite suppression but also on the psychological, behavioral, social, and environmental context of the patient.

Incretin-based pharmacotherapies have changed the clinical management of obesity by producing substantial reductions in body weight and improvements in cardiometabolic risk. However, long-term treatment success depends not only on pharmacological efficacy, but also on neurobehavioral and lifestyle-related factors that influence eating behavior, treatment adherence, and weight-loss maintenance [66,164]. These therapies act partly through central pathways involved in appetite, satiety, food reward, and eating control, but eating behavior is also shaped by mood, stress, sleep, PA, dietary patterns, food environment, stigma, and mental health [165,166]. Therefore, pharmacological weight loss should be interpreted within a broader biopsychosocial framework that integrates biological, behavioral, psychological, and lifestyle determinants of obesity care, as shown in Figure 4. Beyond their peripheral metabolic effects, GLP-1–based medications also act on central nervous system pathways involved in appetite regulation, satiety, food reward, and food-cue reactivity [167]. Neuroimaging studies suggest that GLP-1 receptor agonism may reduce activation in brain regions involved in reward processing and responses to high-calorie food cues, thereby attenuating craving and hedonic eating drive [168]. These effects indicate that the therapeutic action of incretin-based agents is not limited to passive CR, but also involves neurobiological mechanisms that influence eating behavior [169]. However, functional MRI findings should be interpreted primarily as mechanistic evidence, and further longitudinal studies are needed to determine whether these neural changes translate into sustained behavioral adaptation and long-term weight-loss maintenance.

Accordingly, the following sections examine neurocognitive regulation of eating behavior during obesity pharmacotherapy, the role of PA, nutrition, and mental health as lifestyle modulators, and the need for a personalized biopsychosocial model of long-term pharmacological weight management.

### 6.1. Neurocognitive Regulation of Eating Behavior in Obesity Pharmacotherapy

Recent advances in obesity pharmacotherapy suggest that incretin-based agents may influence eating behavior through neurocognitive mechanisms involved in executive control, inhibitory control, reward sensitivity, food-cue reactivity, and food-related decision-making [170,171]. These domains are clinically relevant because eating behavior is not determined only by peripheral satiety signals, but also by cognitive control, emotional state, learned food responses, and exposure to highly palatable food environments [172,173]. In this framework, GLP-1 receptor agonists and dual or multi-receptor agonists may contribute to weight reduction not only by reducing appetite and energy intake, but also by modulating neurobehavioral processes that shape food choice, craving, impulse control, and eating regulation.

Emerging evidence from neurophysiological and behavioral studies indicates that incretin-based therapies may improve inhibitory control over food intake, particularly in environments characterized by highly palatable food exposure [174]. This suggests a potential strengthening of executive control mechanisms, which are primarily mediated by prefrontal cortical networks responsible for self-regulation and decision-making. Such effects may contribute to improved adherence to reduced-energy intake patterns observed during treatment; however, direct evidence linking neurocognitive changes to long-term treatment adherence remains limited [175].

In addition, GLP-1 receptor activation has been associated with altered neural responses to external food cues [176,177]. This may reflect a gradual recalibration of learned eating behaviors, particularly those driven by environmental triggers rather than physiological hunger [178]. However, this should be interpreted as a possible mechanism rather than an established long-term treatment effect, and further longitudinal studies are needed to determine whether changes in food-cue reactivity translate into sustained behavioral adaptation during pharmacological treatment [179].

Importantly, there was also some degree of variability in the neurocognitive response, suggesting that there could be inter-individual differences in the way people eat, how they respond to stress, or how well they control their thoughts and behaviors that could affect treatment outcomes [180]. This highlights the importance of combining pharmacological interventions with behavioral interventions to enhance self-regulation and promote adaptive eating patterns [181]. Together, these mechanisms suggest that sustained weight management may depend not only on pharmacological appetite suppression, but also on behavioral strategies that strengthen self-regulation and food-related decision-making [182].

### 6.2. Lifestyle Modulators of Eating Behavior: Physical Activity, Nutrition, and Mental Health

Although the metabolic effects of PA and nutrition have been discussed in the preceding sections, both may also interact with psychological and behavioral factors during pharmacological weight loss [183,184]. Regular physical activity may support mood regulation, stress management, cognitive flexibility, and self-efficacy, whereas adequate nutritional status and higher diet quality may contribute to behavioral stability and psychological well-being [185,186,187,188]. These interactions provide a relevant context for considering mental health as a determinant of treatment adherence, eating behavior, and long-term weight-loss maintenance.

Mental health is a key determinant of obesity treatment response. Meta-analytic evidence suggests that the odds of depression and anxiety are significantly greater for those with obesity than for normal-weight people, with pooled estimates suggesting a 1.5-fold increased odds [189,190]. Emotional eating, common amongst almost one-third of people with obesity, also can disrupt self-regulation and increase reward-related eating [191]. These factors may interfere with adherence to pharmacological treatment and reduce the sustainability of weight-loss outcomes.

Psychiatric safety should also be considered when evaluating incretin-based therapies. Current observational evidence has not consistently shown an increased risk of psychiatric adverse events with GLP-1–based medications, although further monitoring remains warranted, particularly in individuals with pre-existing mental health conditions [191,192]. Some studies have also reported modest improvements in emotional eating patterns and quality-of-life measures, but psychological responses may vary substantially across individuals, especially among patients with high stress reactivity or low executive control [182,193].

The results are congruent with an integrated biopsychosocial approach to treatment, and indicate that pharmacotherapy alone is not enough for achieving optimal long-term management [56]. Structured physical activity may support mood regulation and self-efficacy; nutritional counseling may improve dietary structure and treatment adherence; and psychological interventions, including cognitive behavioral therapy and stress-management training, may help reduce emotional eating, strengthen impulse control, and support relapse prevention [181,185].

Overall, an integrated approach addresses both metabolic and psychosocial determinants of obesity and may support more sustainable long-term treatment outcomes.

### 6.3. Toward a Personalized Biopsychosocial Model of Long-Term Pharmacological Weight Management

Pharmacological efficacy, lifestyle behaviors, and mental health are all in dynamic interaction, further highlighting the clinical need for a personalized biopsychosocial approach to obesity care [194]. Incretin-based pharmacotherapies offer significant weight loss, but the ultimate success of this treatment will come from the ability to produce long-term functional, psychological, and behavioral improvements [56]. Thus, the outcomes of obesity treatment should go beyond simply assessing the percentage body weight lost, and should be systematically integrated to assess treatment adherence, body composition maintenance, physical functioning, health-related quality of life (HRQoL), emotional health, and long-term maintenance of adaptive eating behaviors [195,196].

Personalization is particularly important because individuals with overweight or obesity differ substantially in baseline phenotype, psychological profile, eating behavior, functional capacity, and treatment expectations [197,198]. Psychological factors such as emotional eating and impaired self-regulation are associated with stress-related overeating and may influence long-term weight maintenance [199,200]. These factors suggest that some patients may require more intensive behavioral support, psychological counseling, or stress-management strategies alongside pharmacotherapy.

Clinical phenotype should also guide treatment personalization. Older adults, individuals with sarcopenic obesity, and patients with low baseline physical function may be more vulnerable during the active weight-loss phase, particularly when lean mass accounts for a meaningful proportion of total weight lost. Lean mass may account for approximately 20–40% of total weight loss in some weight-loss interventions, although this varies according to population, intervention, and assessment method [138,146]. In these vulnerable groups, early integration of exercise and nutritional support may be needed to preserve functional capacity, although detailed recommendations regarding resistance training and protein intake are addressed in Section 3 and Section 4.

A personalized approach also requires careful evaluation of patient-reported outcomes (PROMs). In the STEP clinical trials, semaglutide was most commonly associated with gastrointestinal adverse effects including nausea, diarrhea, vomiting, and constipation. In clinical studies and real-world settings, gastrointestinal symptoms are frequently reported among patients treated with GLP-1 receptor agonists, with prevalence varying depending on population and study design [201,202]. These factors, along with long-term anxiety of dependency on medications, cost burden, and ongoing weight stigma, have a significant impact on persistence. In fact, a recent real-world cohort study confirms the differences between real-world and clinical trials in nearly 50–65% of cases, patients stop taking GLP-1 receptor agonists within the first 12 months of starting therapy. The process of documenting quality of life, mental health, and eating behavior as commonplace clinical outcomes is thus essential to connect clinical effectiveness and efficacy with real-life effectiveness [203,204].

In clinical practice, pharmacotherapy should be embedded within a broader long-term care pathway rather than used as an isolated or episodic intervention [205]. This multidisciplinary pathway should integrate medication, structured PA, individual nutrition counseling, and cognitive-behavioral therapy (CBT) [156]. Such integration may provide an early treatment phase in which patients benefit from the appetite- and reward-modulating effects of dual- or triple-incretin agonists, supporting the development of healthier eating patterns, improved self-regulation of food intake, and more sustainable lifestyle habits during the period of maximal pharmacological response [21,206].

In summary, a personalized biopsychosocial approach shifts obesity management from short-term weight reduction toward long-term optimization of metabolic health, physical function, psychological well-being, and treatment sustainability [194]. By targeting biological, nutritional, behavioral, psychological, and social determinants, this model has the potential to improve long-term treatment sustainability, reduce relapse risk, preserve functional capacity, and support more patient-centered outcomes in pharmacological weight management [207,208,209].

## 7. Clinical Outcomes Beyond Body Weight

Although percentage body weight loss is the traditional primary endpoint for clinical trials and real-world assessment of anti-obesity drug therapy, it only presents a partial picture of therapeutic benefit [1,210]. While the incretins have led to unprecedented degrees of weight loss [26,36], the clinical relevance of such changes needs to be understood in the context of a wider multidimensional framework, including metabolic, functional and patient-centered outcomes [13,58,210]. Therefore, obesity treatment should not be evaluated only through body weight and BMI, but also through interconnected domains such as cardiometabolic risk factors, body composition, skeletal muscle function, bone health, physical performance, health-related quality of life, and weight-loss maintenance [31,51,53].

The following sections examine body composition, cardiometabolic outcomes, physical performance, and health-related quality of life as complementary endpoints that may better capture the clinical value of pharmacological weight loss. By integrating evidence from pivotal incretin-based trials, lifestyle interventions, and exercise studies, this section highlights areas of established benefit and unresolved gaps in the assessment of treatment efficacy beyond body weight.

### 7.1. Quality of Weight Loss: Body Composition and Lean Mass Preservation

Weight loss induced by anti-obesity drugs involves changes in adipose tissue, lean tissue, and body water rather than uniform loss across body compartments [26,211]. Meta-analyses of incretin-based therapy data suggest that about 25–33% of weight loss is related to lean tissue mass components, which is similar to that seen with traditional diet therapy, suggesting that lean mass loss is not a drug-specific effect but a general physiological response to negative energy balance [212]. Comparisons across studies should be interpreted cautiously because of differences in participant characteristics, diabetes status, pharmacological agent, treatment duration, magnitude of weight loss, and methods used to assess body composition.

Methodological caution is required because dual-energy X-ray absorptiometry-derived lean mass includes skeletal muscle, organs, connective tissue, and body water and does not directly measure muscle protein, muscle quality, or myofiber integrity [213,214,215,216]. Consequently, reductions in lean mass should not automatically be interpreted as equivalent skeletal muscle loss or functional impairment [213,214,215,216].

Lean-tissue responses to pharmacologically induced weight loss may vary according to baseline body composition, age, metabolic status, and pre-existing sarcopenic risk [212,217,218]. In particular, individuals with low baseline muscle quality or pre-existing sarcopenic risk may experience more pronounced functional consequences even when absolute reductions in lean mass are modest. Therefore, quantitative changes in lean mass should be interpreted alongside qualitative assessments of muscle function and physical performance [219].

Therefore, when feasible, body-composition monitoring should be integrated with functional assessments such as muscle strength, gait speed, chair-stand performance, and mobility. This approach may help distinguish expected changes in fat-free mass from clinically relevant muscle deterioration and identify patients requiring targeted exercise, nutritional support, or closer monitoring. The main domains defining the quality of weight loss during anti-obesity pharmacotherapy are summarized in Table 3.

### 7.2. Metabolic and Cardiometabolic Outcomes

Incretin-based anti-obesity drugs have systemic metabolic effects that extend beyond reductions in body weight and include improvements in glycemic control, blood pressure, lipid profile, insulin sensitivity, hepatic steatosis, inflammatory markers, adipose tissue function, and renal-metabolic parameters [220]. These effects are clinically relevant because obesity is associated with multiple interrelated metabolic disturbances involving adipose tissue, liver, skeletal muscle, vasculature, and the kidney. Importantly, many of these benefits occur in parallel with weight loss and may be partly mediated by reductions in adiposity, particularly visceral and ectopic fat. However, experimental and clinical evidence also suggests that GLP-1 and GIP receptor signaling may contribute to some metabolic effects through mechanisms that are not entirely explained by weight reduction alone [221,222,223]. Therefore, the cardiometabolic benefits of incretin-based pharmacotherapy should be interpreted as the result of both weight-loss–dependent and potentially receptor-mediated mechanisms. In the SELECT trial, semaglutide 2.4 mg reduced major adverse cardiovascular events by 20% in adults with overweight or obesity and established cardiovascular disease without diabetes, providing direct evidence of cardiovascular benefit beyond weight reduction [224].

Beyond major adverse cardiovascular events, recent evidence also suggests that anti-obesity pharmacotherapy may improve obesity-related complications that contribute to cardiometabolic risk. In the SURMOUNT-OSA phase 3 program, adults with moderate-to-severe obstructive sleep apnea and obesity were randomized to tirzepatide, at the maximum tolerated dose of 10 or 15 mg, or placebo for 52 weeks. In participants not using positive airway pressure therapy at baseline, tirzepatide reduced the apnea-hypopnea index by 25.3 events per hour compared with 5.3 events per hour with placebo. In participants using positive airway pressure therapy, the corresponding reductions were 29.3 and 5.5 events per hour. Tirzepatide also improved body weight, hypoxic burden, high-sensitivity C-reactive protein, systolic blood pressure, and sleep-related patient-reported outcomes. These findings indicate that the clinical value of incretin-based pharmacotherapy may extend to obesity-related complications that are closely linked to cardiovascular and metabolic risk [225].

One of the most consistent metabolic improvements, besides weight loss, is insulin sensitivity. Incretin-based drugs reduce fasting insulin levels and improve surrogate markers of insulin resistance, including HOMA-IR (Homeostatic Model Assessment of Insulin Resistance), which are indicators of hepatic insulin resistance, decreased ectopic lipid deposition, and improved adipose tissue function [26,35,162]. These changes likely reflect several interconnected processes, including reduced visceral adiposity, lower ectopic lipid deposition, improved adipose tissue function, and better hepatic insulin sensitivity. In individuals with obesity and insulin resistance, these effects may contribute to improvements in metabolic syndrome components and reduce the overall burden of cardiometabolic risk.

Hepatic metabolism is another clinically relevant target. Incretin-based therapies have been associated with reductions in liver fat content and improvements in liver enzymes, suggesting potential benefit in patients with metabolic dysfunction-associated steatotic liver disease [226,227,228]. These improvements are probably driven in large part by weight loss and reductions in visceral and ectopic fat. Nevertheless, direct or indirect effects on hepatic metabolism, inflammation, insulin signaling, and adipose tissue–liver crosstalk may also contribute. Further studies are needed to clarify the extent to which hepatic benefits are independent of weight loss and whether they translate into sustained histological improvement in metabolic dysfunction-associated steatohepatitis.

Concurrently, these agents appear to exert beneficial effects on systemic inflammation and on adipokine balance. Decreases in inflammatory markers, including high-sensitivity C-reactive protein, interleukin-6, and tumor necrosis factor alpha, have been reported, suggesting attenuation of chronic low-grade inflammation associated with obesity [229,230]. Improvements in adipose tissue endocrine function may also be reflected by increased adiponectin levels and reduced leptin concentrations, although these changes should be interpreted in relation to the magnitude of fat-mass reduction and baseline metabolic phenotype. These anti-inflammatory and adipose tissue–modulating effects are clinically relevant because chronic low-grade inflammation, endothelial dysfunction, and altered adipokine signaling contribute to atherosclerosis and long-term cardiovascular risk [230,231,232].

Other metabolic benefits include those pertaining to renal and uric acid physiology. Based on preliminary data, incretin-based agents are a promising therapeutic class that may slow the progression of albuminuria and may help stabilize renal function in high-risk patients, potentially through improvements in insulin sensitivity, natriuresis, and reductions in glomerular hyperfiltration [233,234,235]. There is additionally evidence of a reduction in serum uric acid levels, which decreases the risk of gout and positively influences renal metabolic outcomes in susceptible populations [236].

Together, these findings suggest that the cardiometabolic effects of the anti-obesity medications within the incretin class extend beyond HbA1c, blood pressure and lipid profiles. Therefore, they represent a more systemic metabolic modulation involving multiple organ systems such as adipose tissue, liver, vasculature and the kidney [10,237].

### 7.3. Functional Capacity, Physical Performance, and Health-Related Quality of Life

Functional capacity and physical performance are clinically meaningful outcomes in obesity management because they indicate whether weight loss translates into improved daily functioning, mobility, independence, and health status [49]. While weight loss and improvement of cardiometabolic risk factors are therapeutic goals and important findings, they do not fully reflect the patient experience of anti-obesity pharmacotherapy [219,238]. Overweight and obese individuals often have poorer exercise capacity, lower gait efficiency, joint pain, fatigue, decreased cardiorespiratory fitness, and activity limitations [239]. Therefore, the assessment of anti-obesity therapy should not be limited to body weight, but should also consider whether patients are able to move more easily, perform daily activities with less difficulty, and experience better physical well-being [240].

There are several indirect and direct mechanisms by which incretin-based anti-obesity pharmacotherapies can affect functional status. Significant amounts of fat loss lead to decreased mechanical load on weight-bearing joints, increased mobility, and less dyspnea during exertion [241]. Simultaneously, improvements in glycemic control, blood pressure, systemic inflammation, and vascular metabolism further contribute to enhanced energy levels and exercise tolerance [242]. However, weight loss may facilitate functional improvement but does not guarantee it. Functional gains depend on several factors, including preservation of skeletal muscle function, baseline fitness, physical activity levels, pain, joint disease, nutritional adequacy, and the extent of functional lean-tissue loss. Therefore, even clinically meaningful weight loss may result in only modest improvements in strength, endurance, balance, or functional reserve if it is accompanied by physical inactivity, inadequate protein intake, or loss of functional lean tissue [51]. This is especially important in older patients, those with sarcopenic obesity, and patients with poor fitness levels since maintaining muscle function may be more important than the absolute loss of body weight.

Physical performance outcomes can thus be used as a useful measure of the functional effect of pharmacologically induced weight loss. Walking speed, chair stand, grip strength, stair climbing, six-minute walk distance and peak oxygen uptake are some parameters that can provide clinically useful data and which are not obtained by BMI or body weight alone. These outcomes are particularly relevant as skeletal muscle function, cardiorespiratory fitness and mobility are important measures of independence, frailty risk, long-term disability and mortality. In this context, the functional test could help to identify patients who may need more intense exercise prescription or nutritional support or rehabilitation-based interventions during anti-obesity drug therapy [243].

Exercise-based evidence indicates that combining weight reduction with structured training produces more favorable functional outcomes than weight loss alone [244,245,246]. The specific roles and limitations of aerobic and resistance exercise during incretin-based pharmacotherapy are discussed in Section 4.

Another key patient-centered outcome is health-related quality of life. The consequences of obesity include diminished physical function, social engagement, emotional health, body image, sleep, work productivity, and self-esteem. In a number of clinical trial settings, weight loss using incretin-based therapies has been shown to improve weight-related quality of life and physical functioning, especially when there is a substantial amount of body weight loss resulting in improvement of mobility, pain, and perceived health status [247,248,249]. However, quality-of-life responses to GLP-1 receptor agonist therapy are heterogeneous. Some patients may experience improvements in confidence, activity levels, social participation, and physical well-being, whereas others may report treatment-related burdens related to gastrointestinal adverse effects, fear of weight regain, concerns about long-term medication dependence, changes in body image, treatment costs, or weight-related stigma [204,250,251,252].

Therefore, HRQoL must be understood as a multidimensional construct and not just a direct result of weight loss [247,253]. Improvement in physical function may require a decrease in mechanical burden and improvement in cardiometabolic health, and semaglutide trials have shown consistent improvements in physical-function and weight-related quality-of-life domains [248,254]. Thus, successful pharmacological obesity management should be evaluated not only by weight loss, but also by improvements in physical function, independence, well-being, and treatment burden. A lower body weight is clinically meaningful, but its value is greater when accompanied by improved health status, functional capacity, and quality of life.

## 8. Long-Term Maintenance and Prevention of Weight Regain

Long-term weight-loss maintenance remains one of the major challenges in obesity treatment [255]. Contemporary anti-obesity medications, particularly incretin-based therapies such as semaglutide and tirzepatide, can induce substantial weight reduction [256]. However, obesity remains a chronic and relapsing disease, and treatment discontinuation, interruption, or dose reduction may be followed by weight regain because the biological, behavioral, and environmental drivers of obesity are not permanently reversed by initial weight loss [257].

Weight regain is not unique to a specific medication or intervention. It has been observed after lifestyle-based weight loss, pharmacological treatment, and other therapeutic approaches across different populations and study designs [258,259]. This suggests that regain largely reflects the chronic biology of obesity and the physiological adaptations to weight loss, rather than failure of a single drug or treatment strategy. Therefore, weight-loss outcomes achieved during clinical trials should not be interpreted as necessarily permanent, but as responses that usually require long-term therapeutic planning, including continued pharmacotherapy when appropriate and structured non-pharmacological support [57].

This issue has important clinical implications. Most discussions of novel anti-obesity medications focus on the magnitude of weight loss achieved during active treatment, whereas less attention is often given to what happens when treatment is discontinued, reduced, or interrupted because of cost, access barriers, drug shortages, adverse events, tolerability issues, or clinical decisions [260]. Evidence from semaglutide and tirzepatide withdrawal studies indicates that the post-treatment period is a critical determinant of long-term therapeutic success [57]. For this reason, maintenance strategies should be planned during active treatment rather than introduced only after weight regain has occurred [261].

Two aspects are particularly relevant. First, the biological and behavioral mechanisms underlying weight regain must be considered, including increased appetite, reduced satiety, adaptive reductions in energy expenditure, changes in leptin and other appetite-regulating hormones, and alterations in adipose tissue metabolism [262,263,264]. Second, the contribution of lifestyle medicine, behavioral support, long-term follow-up, and combined pharmacological and non-pharmacological strategies should be evaluated as part of chronic obesity care [265].

Available evidence suggests that supervised exercise and structured lifestyle support may help sustain weight loss after pharmacological treatment, although their effectiveness varies across individuals and longer-term studies in diverse populations are still needed [266,267].

Therefore, obesity care should move beyond the short-term achievement of weight loss and place greater emphasis on maintenance, relapse prevention, and preservation of metabolic and functional health. Understanding the mechanisms of weight regain and identifying effective maintenance strategies are essential for making pharmacological obesity treatment durable and clinically meaningful.

### 8.1. Weight Regain After Pharmacological Discontinuation

Withdrawal studies consistently show partial weight regain after discontinuation of incretin-based pharmacotherapy. In the STEP 1 extension, participants regained approximately two-thirds of their previous weight loss within one year after semaglutide withdrawal, together with partial reversal of cardiometabolic improvements [56]. The same trend was observed in the case of tirzepatide. After 36 weeks of treatment, patients who were switched to placebo regained an average of 14.0% body weight during the following year. Patients who continued tirzepatide, on the other hand, shed an additional 5.5% body weight. At the end of follow-up, patients who continued treatment lost 25.3% of the weight from baseline, compared with 9.9% for patients who discontinued treatment [57]. These findings indicate that continued treatment may be required for many patients to maintain the full magnitude of pharmacologically induced weight loss.

Weight regain after weight loss should not be interpreted simply as poor discipline or lack of willpower. It is strongly influenced by biological adaptations that occur after weight reduction. Following weight loss, total energy expenditure may decrease beyond what would be expected from the reduction in body size, a process commonly referred to as adaptive thermogenesis or metabolic adaptation [141,268]. At the same time, appetite-regulating signals may shift in a direction that favors weight regain, with reductions in satiety-related signals and increases in hunger drive. These adaptations can increase appetite, reduce fullness, and promote stronger responses to energy-dense foods, making long-term weight-loss maintenance biologically challenging [258].

Discontinuation of incretin-based therapy may further intensify these pressures by removing pharmacological support for appetite regulation. GLP-1 receptor agonists and dual GIP/GLP-1 receptor agonists reduce appetite, increase satiety, improve eating control, and may reduce food cravings and food-cue reactivity through central and peripheral mechanisms [165,269]. When treatment is discontinued, these appetite- and reward-modulating effects may diminish, allowing hunger, cravings, and responsiveness to food cues to re-emerge in some individuals [270,271,272]. However, individual responses vary, and the relative contribution of appetite, energy expenditure, eating behavior, and environmental exposure to post-treatment weight regain remains insufficiently characterized.

Changes in body composition may also contribute to the difficulty of maintaining weight loss. Substantial weight reduction may be accompanied by decreases in fat-free mass, including lean tissue components, which can contribute to lower resting energy expenditure and reduced metabolic reserve [273,274,275]. Although lean mass measured by DXA does not correspond exclusively to skeletal muscle, preservation of lean tissue and muscle function remains clinically important because it supports strength, mobility, physical performance, and long-term energy expenditure. This provides a rationale for integrating resistance training and adequate protein intake during active pharmacological weight loss, rather than waiting until the maintenance phase.

In real-world practice, discontinuation is frequently unplanned and may result from cost, limited insurance coverage, drug shortages, adverse events, treatment fatigue, or patient preference [274,275,276]. When dose reduction or withdrawal is anticipated, clinicians should reinforce nutritional care, physical activity, behavioral support, and longitudinal monitoring. Patients should also be informed before treatment initiation that weight regain may occur after discontinuation and does not indicate personal failure.

### 8.2. Lifestyle Strategies for Long-Term Weight-Loss Maintenance

In addition to knowing why individuals regain weight when they discontinue weight-loss drugs, another important clinical question is this: Which are the non-drug, structured approaches that can support long-term weight maintenance? Collectively, the available evidence suggests that weight loss achieved with pharmacological treatment is frequently difficult to maintain after treatment discontinuation. Establishing sustainable lifestyle routines during active treatment and continuing them thereafter may support long-term weight maintenance, although direct evidence, particularly for newer incretin-based therapies, remains limited.

Follow-up evidence from the Copenhagen trial suggests that supervised exercise initiated during liraglutide-supported weight loss may improve post-treatment weight and body-composition maintenance [266]. Additional analyses showed that exercise maintained weight loss without increasing appetite or sedentary behavior, while combined exercise and liraglutide improved cognitive restraint and reduced sedentary time during the active maintenance phase [277]. However, these findings were obtained with liraglutide and should not be directly generalized to semaglutide, tirzepatide, or newer agents.

Nutrition is also central during the maintenance phase. When medication dose is reduced or discontinued, appetite may increase, and previous dietary patterns may re-emerge, particularly in obesogenic environments [278,279]. Therefore, dietary strategies should focus on sustainable eating patterns, adequate protein intake, fiber-rich foods, dietary variety, and reduced reliance on ultra-processed foods, added sugars, and excess saturated fat. Adequate protein intake, particularly when combined with resistance training, may support lean-tissue preservation and help attenuate unfavorable changes in resting energy expenditure [280]. A healthy diet should also limit ultra-processed foods, saturated fats, and added sugars and include more vegetables, legumes, whole grains, and lean protein. Such a diet may help maintain metabolic health, as well as psychological health [281]. Adequate protein combined with resistance training may support lean-tissue preservation, although its specific effect on post-incretin weight maintenance remains uncertain.

The third crucial, yet underutilized aspect of long-term maintenance is behavioral and psychological care. Cognitive behavioral therapy for obesity combines behavioral techniques, such as self-monitoring, goal setting, stimulus control, and relapse prevention, with cognitive restructuring. This enables the patient to identify and change thoughts that can interfere with adherence. Dalle Grave et al. suggest that the failure to address these thoughts may be one reason why biological and behavioral treatments for weight loss are not effective at maintaining weight loss over time [282]. In 2021, Iłowiecka et al. performed a randomized controlled study in adults with obesity, demonstrating that an 18-month support program with cognitive behavioral therapy, motivational interviewing, nutritional education, and physical activity assessment was effective in maintaining body weight and anthropometric parameters after a 12-month structured weight-loss program [283]. These findings suggest that early and ongoing behavioral support may facilitate long-term weight maintenance. However, additional studies are needed to determine the optimal timing, intensity, and effectiveness of these interventions in patients treated with newer incretin-based therapies.

Self-monitoring may also support maintenance when used in a patient-centered manner. Monitoring of body weight, food intake, physical activity, sedentary behavior, or symptoms can improve awareness and facilitate early intervention, and digital tools may support this process in some patients [284,285,286,287]. Many patients may discontinue treatment, so it can be helpful to have activity trackers, diet-tracking apps, and periodic weighing as part of standard obesity management. These tools are scalable and may facilitate self-monitoring and long-term engagement in healthy behaviors, although their specific contribution to maintaining weight loss following incretin-based therapy requires further investigation [288,289].

Sleep quality and stress should also be considered within individualized weight-maintenance planning, as discussed in Section 6. In fact, these have an impact on appetite, energy storage through cortisol, and behavior regulation [290].

Overall, the available evidence supports a shift in clinical perspective: anti-obesity medications should not be viewed solely as tools to achieve weight reduction, but also as part of a broader therapeutic window in which sustainable lifestyle changes can be established. Long-term weight maintenance is likely to benefit from an integrated approach combining pharmacotherapy, when appropriate, with physical activity, nutritional care, behavioral support, self-monitoring, and longitudinal follow-up. However, the relative contribution of each component remains to be fully established.

### 8.3. Implementation Challenges and Health-System Considerations

Implementing integrated obesity care remains challenging because access to multidisciplinary services may be limited by workforce shortages, insufficient specialist training, constrained resources, and fragmented referral pathways. Consequently, lifestyle co-therapy may frequently be delivered as brief or generic counseling rather than as a structured and individualized intervention [291].

Economic and health-system barriers may also limit access to integrated obesity treatment. The combined costs of medication, clinical visits, dietetic counseling, supervised exercise, psychological support, and body-composition or functional assessments may be substantial. Access also varies across healthcare systems according to drug reimbursement policies, availability of multidisciplinary services, and access to qualified exercise and nutrition professionals. Consequently, patients in resource-limited settings or with fewer socioeconomic resources may have reduced access to both pharmacotherapy and structured lifestyle support, potentially compromising treatment uptake, adherence, and long-term maintenance and widening existing health inequalities [292]. From a public-health perspective, equitable implementation strategies should therefore accompany the expanding use of anti-obesity medications. From a public-health perspective, equitable implementation strategies should accompany the expanding use of anti-obesity medications. Providing patients with structured education, nutritional counseling, physical activity guidance, symptom management, and appropriate follow-up may help support long-term treatment, although the optimal content and intensity of these interventions have not yet been clearly established. More intensive multidisciplinary care may be particularly appropriate for individuals with persistent gastrointestinal symptoms, nutritional inadequacy, sarcopenic obesity, functional impairment, psychological distress, disordered eating, multiple comorbidities, or a high risk of treatment discontinuation and weight regain. More intensive multidisciplinary care may be prioritized for individuals with persistent gastrointestinal symptoms, nutritional inadequacy, sarcopenic obesity, functional impairment, psychological distress, disordered eating concerns, multiple comorbidities, or a high risk of treatment discontinuation and weight regain.

Future trials should assess the clinical efficacy, feasibility, acceptability, long-term adherence, and cost-effectiveness of integrated lifestyle co-therapy in routine care. From a public-health perspective, equitable implementation strategies should accompany the expanding use of anti-obesity medications across different healthcare settings.

## 9. Conclusions

Incretin-based pharmacotherapies have substantially expanded the treatment options available for obesity. Direct evidence from pivotal trials supports clinically meaningful weight loss and improvements in several cardiometabolic outcomes with semaglutide and tirzepatide [26,31,36]. Body-composition analyses indicate preferential fat-mass reduction accompanied by variable decreases in lean mass [26,46], whereas withdrawal studies demonstrate weight regain and partial reversal of cardiometabolic benefits after treatment discontinuation [56,57]. These findings support evaluating treatment success beyond body weight and BMI by considering body composition, physical function, nutritional adequacy, tolerability, psychological well-being, and long-term maintenance.

In contrast, direct evidence evaluating structured lifestyle co-therapy during treatment with newer incretin-based agents remains limited. The most relevant randomized evidence combining supervised exercise with a GLP-1 receptor agonist derives from liraglutide-based studies and should not be directly generalized to semaglutide, tirzepatide, or newer multi-receptor agonists [69]. Pivotal trials of these newer agents generally included lifestyle counseling but did not isolate structured exercise or specific nutritional interventions as independent treatment components. Recommendations regarding aerobic and resistance exercise, protein intake, diet quality, micronutrient adequacy, hydration, and daily movement are therefore based mainly on indirect evidence from diet-induced weight loss, general obesity care, and expert guidance [12,13,16,60]. The hypothesis that these strategies improve the functional quality and durability of weight loss during newer pharmacotherapies is biologically and clinically plausible but has not yet been conclusively demonstrated.

Accordingly, lifestyle strategies should be interpreted as evidence-informed clinical considerations rather than as agent-specific protocols. Individualized assessment may help identify patients at increased risk of lean-tissue loss, nutritional inadequacy, functional decline, gastrointestinal intolerance, or treatment discontinuation. However, implementation may be constrained by costs, reimbursement policy, limited access to multidisciplinary professionals, and differences across various healthcare systems, potentially contributing to unequal treatment uptake, adherence, and long-term outcomes [291,292].

Overall, this review suggests that treatment evaluation in the era of incretin-based pharmacotherapy should extend beyond the magnitude of weight loss to include physical function, nutritional adequacy, cardiometabolic health, treatment burden, and long-term maintenance. Structured physical activity, nutritional care, and behavioral support may complement pharmacotherapy and contribute to these broader outcomes. The precise contribution of each lifestyle component during newer pharmacological treatments, however, has not yet been conclusively demonstrated; dedicated long-term trials are therefore needed.

## Figures and Tables

**Table 1:** Incretin-based and emerging anti-obesity pharmacotherapies and their efficacy, mechanisms, and lifestyle-relevant considerations.

Therapeutic Approach	Principal Targets and Mechanisms	Representative Clinical Evidence	Lifestyle-Relevant Implications and Unresolved Issues
GLP-1 receptor agonists: liraglutide 3.0 mg; semaglutide 2.4 mg	GLP-1 receptor activation supports satiety, reduces appetite and energy intake, slows gastric emptying, and improves glucose regulation.	Liraglutide and semaglutide produce clinically meaningful weight loss when combined with lifestyle intervention. STEP 1 showed greater weight loss with semaglutide 2.4 mg than placebo. SELECT showed a reduction in major adverse cardiovascular events in adults with overweight/obesity and established cardiovascular disease without diabetes.	Appetite suppression and gastrointestinal symptoms may reduce dietary variety, protein intake, hydration, and micronutrient adequacy. Long-term care may include nutritional support, resistance training, physical activity planning, and strategies to reduce discontinuation and weight regain.
Dual GIP/GLP-1 receptor agonist: tirzepatide	Co-agonism at GIP and GLP-1 receptors enhances appetite regulation, glucose-dependent insulin secretion, and metabolic control.	SURMOUNT-1 showed dose-dependent weight loss. SURMOUNT-3 showed additional weight loss after an intensive lifestyle lead-in. SURMOUNT-5 showed greater reductions in body weight and waist circumference with tirzepatide than semaglutide.	Larger weight reductions increase the need to monitor body composition, muscle strength, physical function, dietary adequacy, and weight-maintenance behaviors. Main issues include gastrointestinal tolerability, cost/access, long-term persistence, and weight regain after withdrawal.
Triple GIP/GLP-1/glucagon receptor agonist: retatrutide, investigational	Triple receptor agonism may influence appetite regulation, glycemic control, lipid metabolism, and energy expenditure.	Phase 2 obesity data showed marked body-weight reduction, with up to 24.2% mean loss at 48 weeks. Longer-term confirmatory evidence remains pending.	The potential for very large weight loss makes lean-tissue preservation, nutritional adequacy, exercise capacity, heart-rate monitoring, and tolerability key outcomes for future studies. Retatrutide is not yet approved for obesity treatment.
Amylin analogue plus GLP-1 receptor agonist: cagrilintide plus semaglutide, CagriSema	Combined amylin and GLP-1 pathway activation enhances satiety, reduces meal size and energy intake, and may further delay gastric emptying.	In REDEFINE 1, CagriSema produced a mean body-weight reduction of 20.4% at 68 weeks, compared with 3.0% with placebo, according to the treatment-policy estimand.	Marked appetite suppression may require proactive protein planning, nutrient-dense food selection, hydration, and gastrointestinal symptom-adapted eating. Long-term persistence, comparative effectiveness, cost, body composition, and functional outcomes still require clarification.
Oral small-molecule GLP-1 receptor agonist: orforglipron / Foundayo	Non-peptide oral GLP-1 receptor agonism, with effects broadly consistent with GLP-1 receptor activation and oral delivery rather than injection.	In ATTAIN-1, orforglipron produced a mean body-weight reduction of 11.2% at 72 weeks at the highest dose, compared with 2.1% with placebo, according to the treatment-regimen estimand. Orforglipron was subsequently approved by the FDA for chronic weight management in eligible adults, in combination with a reduced-calorie diet and increased physical activity.	Oral delivery may improve acceptability and access, but lifestyle co-therapy may be clinically relevant. Main issues include gastrointestinal adverse events, dose optimization, persistence, comparative effectiveness, and long-term body-composition and maintenance data.

Note: Evidence on pharmacological efficacy is derived from agent-specific clinical trials. However, the lifestyle-related implications presented in this table are primarily based on general obesity literature, studies conducted during diet-induced weight loss, limited evidence involving liraglutide, and expert guidance, unless otherwise specified. They should therefore be interpreted as evidence-informed clinical considerations rather than as lifestyle interventions directly validated with each pharmacological agent.

**Table 2:** Evidence-informed lifestyle co-therapy during anti-obesity pharmacotherapy: physical activity and nutritional considerations.

Domain	Therapeutic Role During Pharmacotherapy	Clinical Focus	Suggested Outcomes to Monitor
Aerobic exercise	Supports cardiorespiratory fitness, insulin sensitivity, vascular function, blood pressure, lipid profile, fatigue tolerance, and weight-loss maintenance.	General guidelines suggest gradual progression toward 150–300 min/week of moderate-intensity aerobic activity or 75–150 min/week of vigorous-intensity activity. Use low-impact options when joint pain, low fitness, dyspnea, fatigue, or severe obesity are present.	Cardiorespiratory fitness, 6 min walk distance, step count, blood pressure, lipids, glucose/HbA1c when indicated, fatigue, adherence.
**Resistance training**	Provides an anabolic stimulus to support muscle strength, physical function, bone health, and lean-tissue preservation during weight reduction.	Two to three sessions per week may be considered when feasible, involving major muscle groups and progressive loading. Include balance and mobility exercises in older adults, frailty risk, sarcopenic obesity, or low baseline function.	Handgrip strength, chair-stand performance, gait speed, mobility, falls risk, body composition when available.
**Daily movement and sedentary behavior reduction**	Helps preserve daily movement habits and targets cardiometabolic risk beyond structured exercise.	Increase daily steps progressively; interrupt prolonged sitting when feasible; add post-meal walking, standing tasks, stairs, active commuting, and short light-intensity activity breaks. Individualize for fatigue, pain, disability, and work context.	Step count, sedentary time, activity breaks, wearable-derived light activity, patient-reported barriers.
**Diet quality and dietary pattern**	Ensures that appetite suppression translates into nutrient-dense intake rather than smaller portions of nutritionally poor foods.	Prioritize vegetables, fruits, legumes, whole grains, lean/high-quality protein sources, unsaturated fats, and minimally processed foods. Adapt fiber and fat load during nausea, bloating, reflux, or early satiety.	Dietary variety, fiber intake, exposure to ultra-processed foods and saturated fat, blood pressure, lipids, glucose/HbA1c when indicated.
**Protein adequacy**	Supports lean tissue, muscle protein synthesis, strength, resting energy expenditure, and the functional quality of weight loss.	Individualize protein intake rather than prescribing a rigid target. Evidence discussed in the text supports approximately 1.2–1.5 g/kg/day during weight loss, with higher targets, around 1.5–2.0 g/kg/day, considered in older adults or patients with sarcopenic obesity when clinically appropriate. Adapt according to kidney function, comorbidities, baseline intake, body composition, energy intake, gastrointestinal tolerance, and resistance training.	Protein intake, meal distribution, renal function when clinically indicated, strength/function tests, body composition where available.
**Micronutrients and hydration**	Reduces risk of inadequacy during lower total intake, reduced dietary variety, gastrointestinal symptoms, or restrictive dietary patterns.	Assess vitamin D, calcium intake, iron, folate, vitamin B12, zinc, magnesium, electrolytes, and renal function according to dietary pattern, symptoms, comorbidities, age, medication use, and clinical risk.	Dietary recall, hydration symptoms, constipation/diarrhea, vitamin D, B12, iron status, folate, electrolytes, renal function as indicated.
**Gastrointestinal symptom-adapted eating**	Improves tolerability, nutrient intake, hydration, exercise tolerance, and medication persistence during treatment initiation and dose escalation.	Use smaller meals, slower eating, lower-fat meals during symptomatic periods, gradual fiber titration, adequate fluids, avoidance of trigger foods, and soft or liquid protein options during nausea or early satiety.	Nausea, vomiting, reflux, bloating, constipation, diarrhea, hydration, protein intake, dose interruptions, discontinuation risk.

**Table 3:** Quality of weight loss during anti-obesity pharmacotherapy: body composition, function, and monitoring.

Domain	Potential Benefits	Key Clinical Concerns	Lifestyle Strategies and Monitoring
Fat mass and visceral adiposity	Incretin-based therapies generally produce substantial fat-mass reduction and improve adiposity-related metabolic risk. In the SURMOUNT-1 DXA substudy, tirzepatide reduced fat mass substantially, and approximately 75% of weight lost was fat mass.	Body weight alone does not indicate whether weight loss is metabolically and functionally favorable.	Combine pharmacotherapy with aerobic activity, resistance training, and high-quality dietary patterns. Monitor weight, waist circumference, body composition when available, and cardiometabolic markers.
Lean mass and fat-free mass	Fat-mass reduction is usually greater than lean-mass loss, but lean mass can decline during major weight reduction. STEP 1 and SURMOUNT-1 body-composition analyses show preferential fat-mass loss with concomitant lean-mass reduction.	Lean-tissue loss may be clinically relevant in older adults, frailty, sarcopenic obesity, or low baseline function. DXA-derived lean mass is not equivalent to skeletal muscle mass.	Prioritize resistance training and adequate protein intake. Monitor body composition, muscle strength, physical function, and dietary protein adequacy.
Muscle strength and physical function	Weight reduction may improve mobility and reduce mechanical burden.	Weight loss alone does not guarantee preservation of strength, balance, or independence. Pivotal pharmacotherapy trials do not consistently measure muscle strength or physical performance.	Include progressive resistance training, balance, and mobility exercises, especially in individuals at higher functional risk. Monitor handgrip strength, chair-stand performance, gait speed, mobility, and falls risk.
Cardiorespiratory fitness and activity capacity	Weight loss may improve physical function, while exercise provides additional cardiovascular and functional benefits.	Patients may lose weight while remaining sedentary or physically deconditioned; improvements in cardiorespiratory fitness are not automatic.	Promote progressive aerobic activity and daily movement. Monitor walking capacity, activity levels, fatigue, blood pressure, and fitness measures when feasible.
Bone health	Weight reduction may improve several obesity-related risks, but musculoskeletal effects require attention during substantial weight loss.	Substantial weight loss, regardless of modality, may influence bone turnover and bone mineral density, especially with low lean mass, poor balance, inadequate calcium/vitamin D intake, older age, or postmenopausal status.	Support resistance and balance training, adequate protein intake, calcium/vitamin D adequacy, and fall-risk reduction when appropriate. Monitor bone health in high-risk individuals.
Patient-centered treatment outcomes	Pharmacotherapy may improve symptoms, mobility, and health-related quality of life in selected obesity-related complications.	Gastrointestinal symptoms, cost, treatment burden, stigma, fear of weight regain, and concerns about long-term therapy may affect treatment success.	Use shared decision-making, symptom management, behavioral support, sleep/stress care, and individualized maintenance planning. Monitor quality of life, fatigue, sleep, mood, adherence, persistence, treatment burden, and patient goals.

Note: Pharmacotherapy-specific evidence supports several body-composition and cardiometabolic outcomes summarized in this table. In contrast, the proposed lifestyle strategies and functional monitoring approaches are largely derived from general obesity care, diet-induced weight-loss studies, limited liraglutide-based evidence, and expert recommendations. Their effectiveness during treatment with semaglutide, tirzepatide, or newer multi-receptor agonists has not yet been established conclusively.

## Data Availability

The raw data supporting the conclusions of this article will be made available by the authors on request.

## Abbreviations

The following abbreviations are used in this manuscript:

BMD	Bone mineral density
BMI	Body mass index
CBT	Cognitive-behavioral therapy
CR	Caloric restriction
DASH	Dietary Approaches to Stop Hypertension
DXA	Dual-energy X-ray absorptiometry
GIP	Glucose-dependent insulinotropic polypeptide
GIP/GLP-1	Glucose-dependent insulinotropic polypeptide/glucagon-like peptide-1
GLP-1	Glucagon-like peptide-1
HbA1c	Glycated hemoglobin A1c
HOMA-IR	Homeostatic Model Assessment of Insulin Resistance
HRQoL	Health-related quality of life
LM	Lean mass
MRI	Magnetic resonance imaging
NEAT	Non-exercise activity thermogenesis
PA	Physical activity
SCFAs	Short-chain fatty acids
SMM	Skeletal muscle mass
WHO	World Health Organization
